# Optimized expression and purification of adipose triglyceride lipase improved hydrolytic and transacylation activities *in vitro*

**DOI:** 10.1016/j.jbc.2021.101206

**Published:** 2021-09-17

**Authors:** Natalia Kulminskaya, Claudia Radler, Roland Viertlmayr, Christoph Heier, Peter Hofer, Mariana Colaço-Gaspar, Raymond J. Owens, Robert Zimmermann, Renate Schreiber, Rudolf Zechner, Monika Oberer

**Affiliations:** 1 Institute of Molecular Biosciences, University of Graz, Graz, Austria; 2 Division of Structural Biology, The Welcome Centre for Human Genetics, University of Oxford, Oxford, UK; 3 Protein Production UK, Research Complex at Harwell, Didcot, UK; 4 BioTechMed, Graz, Austria; 5 BioHealth Field of Excellence, University of Graz, Graz, Austria

**Keywords:** PNPLA2, CGI-58, ABHD5, G0S2, lipolysis, ATGL, adipose triglyceride lipase, BSA, bovine serum albumin, CGI-58, comparative gene identification-58, CV, column volume, DAG, diacylglycerol, DGAT, diacylglycerol acyltransferase, FA, fatty acid, G0S2, G0/G1 switch gene 2, GST, glutathione-*S*-transferase, HEK293T, human embryonic kidney 293T, His6, hexahistidine tag, HSL, hormone-sensitive lipase, LD, lipid droplet, MAG, monoacylglycerol, mATGL, mouse ATGL, MBP, maltose-binding protein, MTBE, methyl *tert*-butyl ether, Ni^2+^, nickel (II) ion, NTA, nitrilotriacetic acid, NusA, transcription termination/antitermination protein, ONC, overnight culture, OPPF, Oxford Protein Production Facility, PNPLA, patatin-like phospholipase, SEC, size-exclusion chromatography, SMT3, small ubiquitin-like modifier, TAG, triacylglycerol, TCEP, Tris(2-carboxyethyl)phosphine, TEV, tobacco etch virus, TRX, thioredoxin

## Abstract

Adipose triglyceride lipase (ATGL) plays a key role in intracellular lipolysis, the mobilization of stored triacylglycerol. This work provides an important basis for generating reproducible and detailed data on the hydrolytic and transacylation activities of ATGL. We generated full-length and C-terminally truncated ATGL variants fused with various affinity tags and analyzed their expression in different hosts, namely *E.coli*, the insect cell line Sf9, and the mammalian cell line human embryonic kidney 293T. Based on this screen, we expressed a fusion protein of ATGL covering residues M1-D288 flanked with N-terminal and C-terminal purification tags. Using these fusions, we identified key steps in expression and purification protocols, including production in the *E. coli* strain ArcticExpress (DE3) and removal of copurified chaperones. The resulting purified ATGL variant demonstrated improved lipolytic activity compared with previously published data, and it could be stimulated by the coactivator protein comparative gene identification-58 and inhibited by the protein G0/G1 switch protein 2. Shock freezing and storage did not affect the basal activity but reduced coactivation of ATGL by comparative gene identification 58. *In vitro*, the truncated ATGL variant demonstrated acyl-CoA–independent transacylation activity when diacylglycerol was offered as substrate, resulting in the formation of fatty acid as well as triacylglycerol and monoacylglycerol. However, the ATGL variant showed neither hydrolytic activity nor transacylation activity upon offering of monoacylglycerol as substrate. To understand the role of ATGL in different physiological contexts, it is critical for future studies to identify all its different functions and to determine under what conditions these activities occur.

All known organisms use fatty acids (FAs) as energy substrates and precursors for membrane and signaling lipids. In mammals, FAs are stored in the form of triacylglycerols (TAGs) predominantly in white adipose tissue. From there, FAs can be mobilized during energy demand in a process termed “neutral” lipolysis, which involves a complex regulatory network utilizing hormones, lipids, proteins, and post-translational modifications. For example, hormonal stimulation by catecholamines causes elevated activity of cAMP-dependent protein kinase A leading to a ∼100-fold increase of FA and glycerol release from adipose tissue (1, 2, 3, 4, 5, 6).

The biochemistry of intracellular lipolysis predominantly applies a sequential series of hydrolytic processes. In the first step, adipose triglyceride lipase (ATGL) hydrolyzes TAG to diacylglycerol (DAG) and an FA. The hydrolytic activity of ATGL is enhanced upon direct protein interaction with the coactivator protein comparative gene identification-58 (CGI-58, also named Abhydrolase domain–containing protein 5 [ABHD5]) (7, 8). Conversely, ATGL activity is inhibited by G0/G1 switch gene 2 (G0S2) and hypoxia-inducible protein 2 (also named hypoxia-inducible lipid droplet–associated protein [HILPDA]) (9, 10). In the second step, hormone-sensitive lipase (HSL) acts as the principal enzyme and hydrolyzes DAG to monoacylglycerol (MAG) and an FA (11). ATGL and HSL in addition exhibit DAG and TAG lipase activities, respectively (12). However, the *in vivo* relevance of these activities is unclear. Together, ATGL and HSL are responsible for more than 95% of the total TAG hydrolytic activity in white adipose tissue (12). In the third step, monoacylglycerol lipase and abhydrolase domain–containing protein 6 hydrolyze MAG to FA and glycerol (13). In addition to the hydrolytic activity, ATGL exerts transacylation activity as reported in a few studies (14, 15). TAG synthesis is usually catalyzed by acyl-CoA: diacylglycerol acyltransferase (DGAT) enzymes, DGAT1 or DGAT2 (16, 17). A very recent study suggests functional interdependencies between ATGL and HSL and shows that ATGL functions as a transacylase when HSL is absent (15). Both lipases also can be inhibited by long-chain acyl-CoAs (18). HSL exhibits transacylation activity and contributes to TAG synthesis from DAG in adipose tissue extracts in the absence of ATGL (15). The authors propose that ATGL and HSL have interchangeable functions of hydrolysis and transacylation, which could generate a certain level of functional resilience in biological species.

ATGL and HSL act at the water–lipid droplet interphase, which requires specialized features. Those include simultaneous interactions with a lipid phase *via* hydrophobic modules and with an aqueous phase, which is commonly attributed to hydrophilic parts. ATGL is predicted to harbor regions of intrinsic disorder, which are often associated with high flexibility and multiple interaction partners (19). Because of these intrinsic features, expression of ATGL in a soluble and an active form has been difficult in heterologous systems. ATGL tends to aggregate and precipitate even at low protein concentrations. In addition, the lipase is difficult to purify and handle because of the high sensitivity of its enzymatic activity to various detergents (20).

Though ATGL has been identified in 2004, a detailed protein purification protocol has not been available (14, 21, 22). Numerous groups started to characterize ATGL using partially purified protein or cellular lysates overexpressing ATGL. In this article, we present for the first time highly active and purified lipase. We optimized the ATGL construct size and tested different expression hosts at the Oxford Protein Production Facility (OPPF-UK) (23). We provide a detailed protocol to purify an active C-terminally truncated variant of ATGL (M1-D288) from the *E.* *coli* strain ArcticExpress (DE3). This purified ATGL variant was used to further investigate the first step of intracellular lipolysis in terms of substrate specificity under basal, CGI-58–stimulated, and G0S2-inhibited conditions. Therefore, we applied *in vitro* activity assays using phospholipid-emulsified lipid substrates and separated the reaction products by TLC, thus confirming the previously reported TAG hydrolytic and transacylation activities (14, 15, 21, 22).

## Results

### Rationale for ATGL truncations and high-throughput cloning of ATGL at OPPF-UK

**Figure 1:**
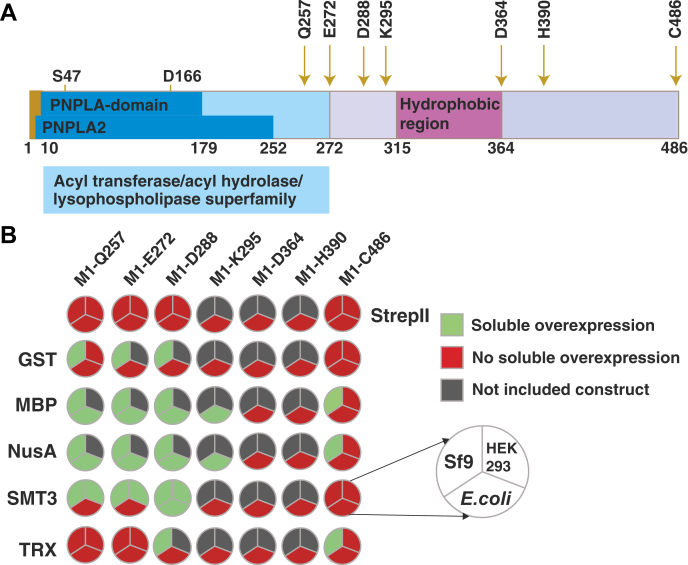
**Overview of ATGL expression with different fusion proteins in different expression hosts.***A*, schematic view of ATGL with domain boundaries according to InterPro and the seven variants tested (*golden arrows*) at the OPPF-UK. The catalytic residues S47 and D166 are indicated. *B*, graphical visualization of summarized results of ATGL variants and their expression in *E.coli*, Sf9, and HEK293T cells. Color code: *green*, soluble expression; *red*: no soluble expression; and *gray*: not tested. See also Table 1 for insoluble expression. ATGL, adipose triglyceride lipase; HEK293T, human embryonic kidney 293T; OPPF, Oxford Protein Production Facility; PNPLA, patatin-like phospholipase domain; PNPLA2, patatin-like phospholipase domain containing protein 2.

For the design of the variants of mouse ATGL (full-length mATGL, M1-Q257, M1-E272, M1-D288, M1-K295, M1-D364, and M1-H390; Fig. 1*A*), we combined structure and domain predictions with experimental data of mouse ATGL and human ATGL. The proteins have 87% overall sequence identity. Regarding sequence conservation, three regions can be distinguished: residues 1 to 266 have 92% identity; residues 298 to 486 have 88% identity; whereas, the intermittent residues 267 to 295 share only 21% identity. According to domain prediction servers, ATGL harbors a patatin-like phospholipase (PNPLA) domain signature within residues 10 to 179, whereas the PNPLA2 region ranges until residue 252, and further extends to residue 272 in the acyl transferases/acyl hydrolases/lysophospholipases homologous superfamily (Fig. 1*A*) (24). The remaining more than 200 residues are mostly disordered or show propensities for α-helices.

In previous work, we showed that C-terminal truncations beyond residue 254 abrogate triglyceride-hydrolytic activity of ATGL (25). ATGL harbors a proline-rich stretch (which is very likely disordered) C-terminal region of Q257, and therefore, we picked the variant ending with Q257 as the shortest variant. The variant stopping after E272 includes this proline-rich region and corresponds to the domain boundary of the homologous superfamily. The variant D288 represents a variant with a premature stop (Q289X) based on a mutation (865C-T) of human patients (26). We selected K295 as additional C-terminal truncation site since (i) it is at the end of the less conserved region that connects the well conserved N-terminal and the conserved C-terminal regions and (ii) residues Arg and Lys represent a clear cut to the preceding disorder promoting glutamic acid–rich stretch, and these residues can potentially engage in electrostatic interactions with the negatively charged stretch. Long helical segments are predicted C-terminal regions of K295. The variant stopping at D364 includes a ca. 50-amino acid long hydrophobic region of ATGL, the last variant ending at H390 includes yet another predicted helical stretch.

During the revision phase of the article, AlphaFold, version 2.0, became publicly available (27) (https://alphafold.ebi.ac.uk/entry/Q8BJ56). The confidence score of the AlphaFold model of mATGL reaches “very high” and “confident” only within residues W8–D197 and H203–L255. According to the program, regions with “low” and “very low confidence rates” might be unstructured in isolation. L320–L387 are predominately helical with a kink in the helix after P362. The regions P258–R297 as well as L391–C486 are predicted only with “very low confidence” and project away from the compacter patatin core domain. Thus, our chosen domain boundaries are completely in line with the latest structure modeling approaches. In sum, we constructed six different C-terminal truncations of mouse ATGL (M1-Q257, M1-E272, M1-D288, M1-K295, M1-D364, and M1-H390) and the full-length protein (M1-C486). Combination with different fusion proteins (glutathione-*S*-transferase [GST], maltose-binding protein [MBP], transcription termination/antitermination protein NusA [NusA], small ubiquitin-like modifier [SMT3], and thioredoxin [TRX]) located N-terminally of the respective ATGL variants resulted in 42 variants tested at the OPPF-UK (Fig. 1 and Table 1).

**Table 1:** List of mouse ATGL variants designed for cloning and expression screening at the OPPF

Fusion	N/C-term amino acid (POI)	Vector (pOPIN)	Design	Molecular weight (kDa)	Host tested	Host expressed
	M1-Q257	pOPINFS	HIS6-3C-POI-StrepII	31	B, I, M	—
	M1-E272	pOPINFS	HIS6-3C-POI-StrepII	33	B, I, M	—
	M1-D288	pOPINFS	HIS6-3C-POI-StrepII	35	B, I, M	—
	M1-K295	pOPINFS	HIS6-3C-POI-StrepII	36	B	—
	M1-D364	pOPINFS	HIS6-3C-POI-StrepII	43	B	—
	M1-H390	pOPINFS	HIS6-3C-POI-StrepII	46	B	—
	M1-C486	pOPINFS	HIS6-3C-POI-StrepII	56	B, I, M	—
GST	M1-Q257	pOPINJ	HIS6-GST-3C-POI	56	B, I, M	(B), I
GST	M1-E272	pOPINJ	HIS6-GST-3C-POI	58	B, I	I
GST	M1-D288	pOPINJ	HIS6-GST-3C-POI	60	B, I	(B), I
GST	M1-K295	pOPINJ	HIS6-GST-3C-POI	60	B	(B)
GST	M1-D364	pOPINJ	HIS6-GST-3C-POI	68	B	—
GST	M1-H390	pOPINJ	HIS6-GST-3C-POI	71	B	—
GST	M1-C486	pOPINJ	HIS6-GST-3C-POI	81	B, I, M	—
MBP	M1-Q257	pOPINM	HIS6-MBP-3C-POI	71	B, I	B, I, (M)
MBP	M1-E272	pOPINM	HIS6-MBP-3C-POI	73	B, I	B, I
MBP	M1-D288	pOPINM	HIS6-MBP-3C-POI	74	B, I	B, I
MBP	M1-K295	pOPINM	HIS6-MBP-3C-POI	75	B	B
MBP	M1-D364	pOPINM	HIS6-MBP-3C-POI	83	B	(B)
MBP	M1-H390	pOPINM	HIS6-MBP-3C-POI	86	B	(B)
MBP	M1-C486	pOPINM	HIS6-MBP-3C-POI	96	B, I, M	I
NUSA	M1-Q257	pOPINNUSA	HIS6-NUSA-3C-POI	85	B, I	B, I
NUSA	M1-E272	pOPINNUSA	HIS6-NUSA-3C-POI	87	B, I	B, I
NUSA	M1-D288	pOPINNUSA	HIS6-NUSA-3C-POI	89	B, I	B, I
NUSA	M1-K295	pOPINNUSA	HIS6-NUSA-3C-POI	89	B	B
NUSA	M1-D364	pOPINNUSA	HIS6-NUSA-3C-POI	97	B	(B)
NUSA	M1-H390	pOPINNUSA	HIS6-NUSA-3C-POI	100	B	(B)
NUSA	M1-C486	pOPINNUSA	HIS6-NUSA-3C-POI	110	B, I, M	I
SMT3	M1-Q257	pOPINS3C	HIS6-SMT3-3C-POI	41	B, I, M	(B), I, M
SMT3	M1-E272	pOPINS3C	HIS6-SMT3-3C-POI	43	B, I, M	(B), I, M
SMT3	M1-D288	pOPINS3C	HIS6-SMT3-3C-POI	45	B, I, M	B, I, M
SMT3	M1-K295	pOPINS3C	HIS6-SMT3-3C-POI	46	B	(B)
SMT3	M1-D364	pOPINS3C	HIS6-SMT3-3C-POI	53	B	(B)
SMT3	M1-H390	pOPINS3C	HIS6-SMT3-3C-POI	56	B	(B)
SMT3	M1-C486	pOPINS3C	HIS6-SMT3-3C-POI	67	B, I, M	(M)
TRX	M1-Q257	pOPINTRX	HIS6-TRX-3C-POI	42	B, I, M	(B), (M)
TRX	M1-E272	pOPINTRX	HIS6-TRX-3C-POI	44	B, I, M	(B)
TRX	M1-D288	pOPINTRX	HIS6-TRX-3C-POI	45	B, I	(B), I
TRX	M1-K295	pOPINTRX	HIS6-TRX-3C-POI	46	B	(B)
TRX	M1-D364	pOPINTRX	HIS6-TRX-3C-POI	54	B	(B)
TRX	M1-H390	pOPINTRX	HIS6-TRX-3C-POI	57	B	(B)
TRX	M1-C486	pOPINTRX	HIS6-TRX-3C-POI	67	B, I, M	I

Variants were tested for overexpression in *E.coli* “B,” the insect cell line Sf9 “I,” and the mammalian HEK293T cells “M.” In the last column, detectable, yet insoluble target protein of the protein is indicted in brackets, for example, (B).

Abbreviations: N/C-term amino acid, first and last amino acid of mATGL in the variant (not including any tags); POI, protein of interest.

### High-throughput overexpression at OPPF-UK

The designed ATGL variants were expressed with different combinations of fusion proteins and affinity tags in the OPIN vector suite (Table 1) to test the production of soluble protein in *E. coli*, insect cells, and mammalian cells at the OPPF-UK (23). We assumed the N-terminal hexahistidine tag (His6) to be accessible for purification purposes when an additional N-terminal fusion protein is present. Only those variants that did not carry any N-terminal fusion protein had a C-terminal StrepII tag for purification (Fig. 1*B*). Overexpression and solubility in *E. coli* were analyzed by SDS-PAGE of (i) protein fractions purified from the soluble supernatant and (ii) protein fractions purified from the insoluble pellet under denaturing conditions. No detectable expression was observed for ATGL variants containing only the small N-terminal His6 tag and a C-terminal StrepII tag. N-terminal addition of GST or TRX fusion protein to the ATGL variants yielded insoluble protein in *E. coli* (Fig. 1*B*). ATGL variants harboring N-terminal MBP, NusA, and SMT3 tags resulted in both soluble and insoluble protein irrespective of the construct size. In total, only nine constructs tested positive for soluble overexpression of ATGL variants in *E. coli*, namely HIS6-MBP-3C-[M1-Q257], HIS6-MBP-3C-[M1-E272], HIS6-MBP-3C-[M1-D288], HIS6-MBP-3C-[M1-K295], HIS6-NusA-3C-[M1-Q257], HIS6-NusA-3C-[M1-E272], HIS6-NusA-3C-[M1-D288], HIS6-NusA-3C-[M1-K295], and HIS6-SMT3-3C-[M1-D288]. About 18 variants produced insoluble protein when tested in bacterial hosts using our experimental conditions (Table 1). Our results indicate that solubility strongly depends on the fusion partners' construct lengths and induction methods. We observed no marked difference between Lemo21 and Rosetta 2 cells. Induction with IPTG resulted in nine soluble variants, whereas autoinduction conditions led to less soluble proteins of the same constructs (data not shown). These data indicate that protein production is possible in *E. coli*, yet that rate of protein production and consequently proper folding might be a limiting factor for the expression of ATGL in its native conformation.

Based on this initial expression screening, the number of variants tested in additional expression systems was reduced to those that at least showed insoluble expression (Table 1). Full-length constructs and the truncations M1-Q257, M1-E272, and M1-D288 fused to an N-terminal His tag were still included as controls to test a potentially negative influence of the fusion proteins upon expression in insect cells or mammalian cells. In agreement with the expression patterns in bacterial host strains, lack of N-terminal fusion proteins did not result in robust (and soluble) protein expression in mammalian cells either. Soluble protein fractions were obtained for 16 of 24 tested ATGL variants harboring N-terminal GST, MBP, NusA, SMT3, or TRX-fusion partners upon expression in Sf9 cells (Fig. 1). Lower virus titer during infection resulted in improved solubility of the target protein. Expression in human embryonic kidney 293T (HEK293T) cells was tested by immunoblotting and revealed no overexpression in the absence of N-terminal fusion proteins and of variants with N-terminal GST and NusA fusion proteins. The analysis of total lysates revealed expression of MBP, SMT3, and TRX variants (Table 1). Only ATGL-fusion variants with SMT3 (HIS6-SMT3-3C-[M1-Q257], HIS6-SMT3-3C-[M1-E272], and HIS6-SMT3-3C-[M1-D288]) resulted in robust overexpression of soluble ATGL variants in HEK293T cells (Fig. 1*B* and Table 1).

Taking together, fusion of mATGL variants with the small protein SMT3 (11.2 kDa) stands out as a prominent lead for ATGL overexpression in different expression systems (Fig. 1*B*). Small cleavable tags are advantageous and less likely to modify activity. Thus, SMT3 tags were considered favorable to reach our aim to purify and characterize ATGL variants *in vitro*. Furthermore, the SMT3 tag had a positive influence in protein expression in all three different expression systems. In case it is needed, the purification protocols developed for SMT3-tagged variants can be adopted to equivalent variants expressed in *E. coli*, Sf9, and mammalian cells system.

### Purification of mATGL288-Str in Lemo21 (DE3) cells and ArcticExpress (DE3) cells yield active protein with residual copurified chaperones

**Figure 2:**
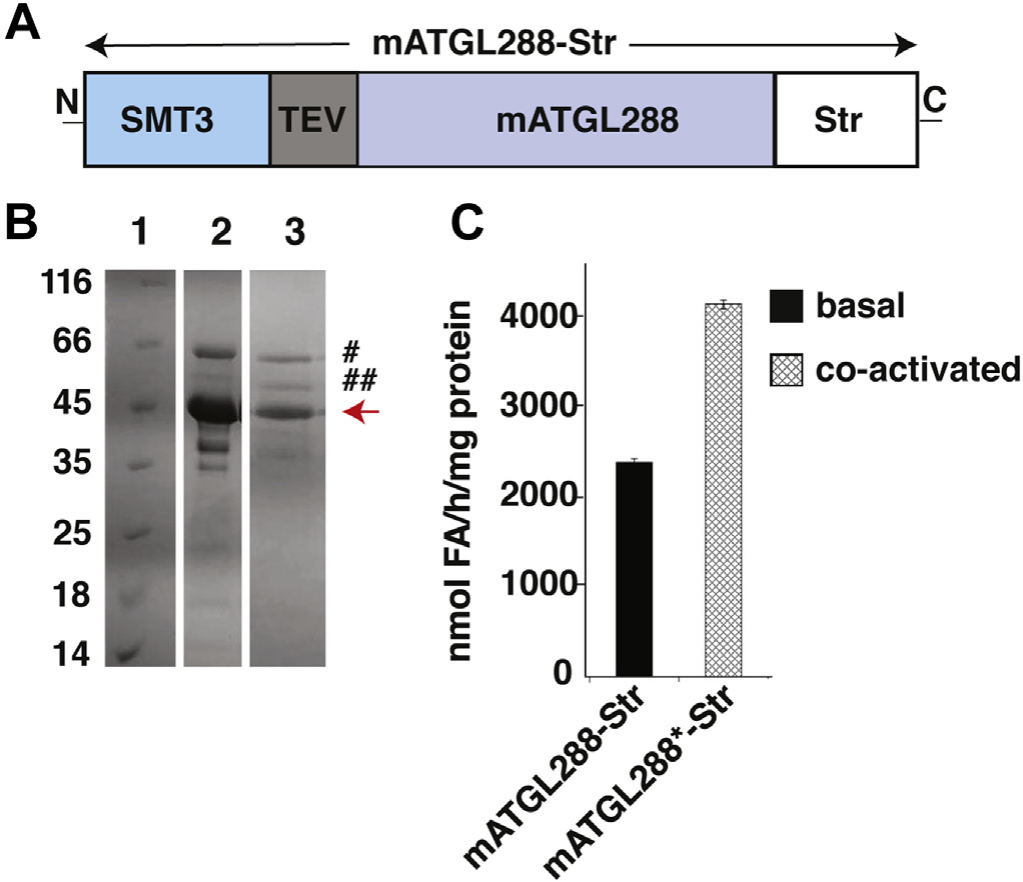
**Purification and activity of mATGL288-Str expressed in Lemo21 (DE3) cells.***A*, schematic drawing of the mATGL288-Str variant. *B*, SDS-PAGE (12%) after affinity purification of mATGL288-Str. Lane 1, Pierce unstained protein MW marker. Lane 2, elution fraction after the StrepII tag purification. Lane 3, elution fraction from size-exclusion chromatography. *Red arrow* indicates mATGL288-Str (45.3 kDa), # and ## indicate DnaK (70.0 kDa) and GroEL (60.0 kDa), respectively. *C*, triacylglycerol hydrolytic activity of purified mATGL288-Str in basal and mCGI-58-stimulated (indicated by an *asterisk*) condition. Str, StrepII tag.

**Figure 3:**
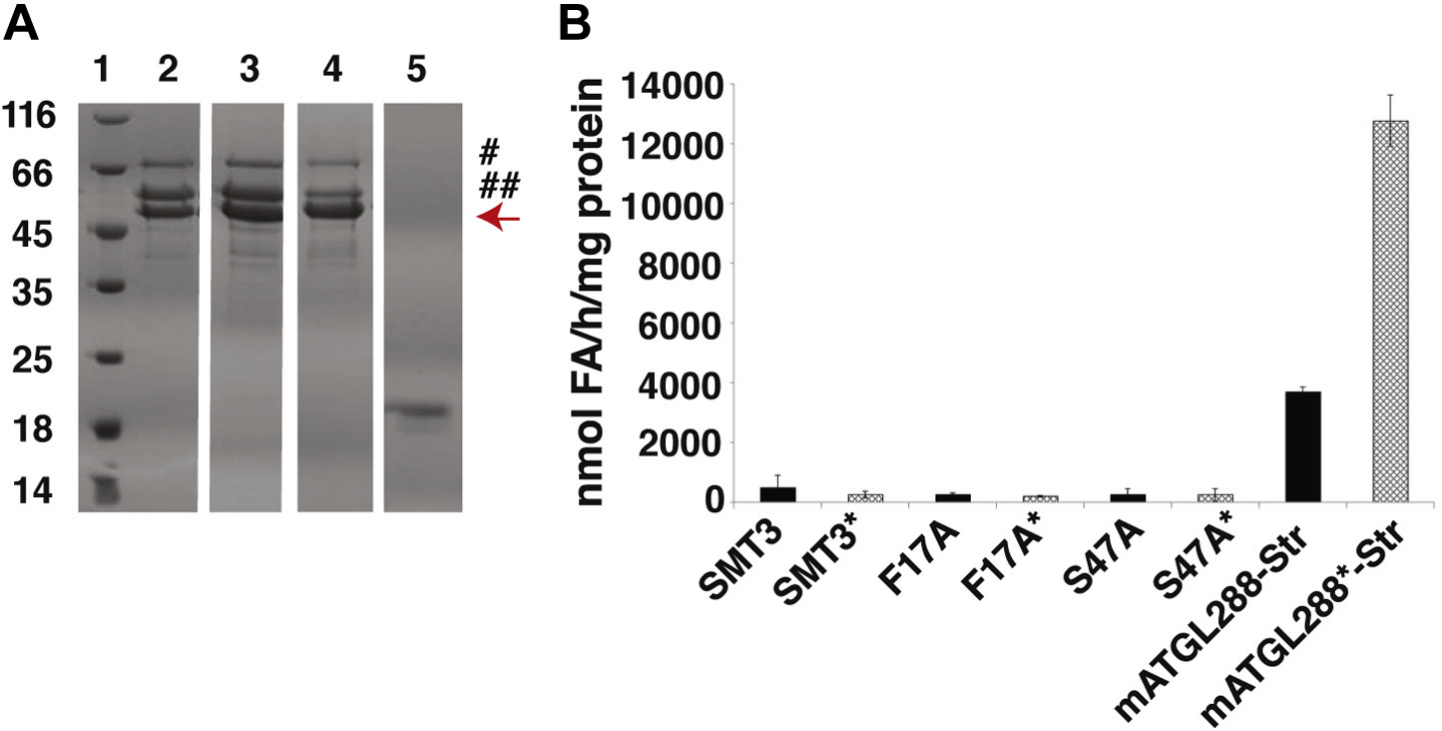
**Purification and activity of mATGL288-Str and variants thereof expressed in ArcticExpress (DE3) cells.***A*, SDS-PAGE after affinity chromatography of mATGL288-Str and the variants F17A and S47A. Lane 1, Pierce unstained protein MW marker. Lane 2, mATGL288-Str. Lane 3, mATGL288 (F17A)-Str. Lane 4, mATGL288 (S47A)-Str. Lane 5, SMT3 protein. # and ## indicate DnaK (70.0 kDa) and GroEL (60.0 kDa), respectively. *B*, triacylglycerol hydrolase activity assay of purified proteins: fusion protein SMT3, control, mATGL288 (F17A)-Str, mATGL288 (S47A)-Str, and mATGL288-Str expressed in ArcticExpress (DE3) cells. The copurified chaperones DnaK and GroEL showed no unspecific triacylglycerol hydrolase activity as tested in the inactive SMT3-TEV-mATGL288 variants F17A and S47A. Coactivation of ATGL with mCGI-58 is indicated by an *asterisk* (∗). ATGL, adipose triglyceride lipase.

Based on our results from the OPPF-UK, we optimized overexpression and purification protocols for an SMT3-tagged mATGL variant truncated after D288 in a bacterial system. The C-terminal tag was chosen to avoid purification of mATGL288, which had undergone potential C-terminal degradation (Fig. 2). This variant can be expressed in different host organisms and should retain TAG hydrolytic activities and regulatory mechanisms *via* protein–protein interaction based on previous studies (10, 25). The expression plasmid encoding the variant mATGL288-Str (Fig. 2*A*) was first expressed in Lemo21 (DE3) cells, which had been used for screening; however, the resulting expression levels were low. SDS-PAGE analysis revealed copurified proteins, which were subsequently identified as DnaK and GroEL by mass spectrometry (data not shown). Both chaperones could not be removed completely during affinity chromatography employing the StrepII tag followed by size-exclusion chromatography (SEC) and SDS-PAGE (Fig. 2*B*). The retention volume during SEC corresponded to a molecular weight of approximately 1050 kDa indicating aggregation, yet the purified protein showed basal activity of ∼2300 nmol FA/(h ∗ mg protein) and could be approximately twofold stimulated with mCGI-58 to ∼4000 nmol FA/(h ∗ mg protein) (Fig. 2*C*). Copurification of chaperones indicates insufficient protein folding. Thus, we tested ArcticExpress (DE3) cells as bacterial expression host that confers better protein processing properties with respect to protein misfolding and insolubility because of cold-adapted chaperonins. Using ArcticExpress (DE3), we expressed the protein at 10 °C and added MgCl_2_ at an early stage to support chaperon removal during purification. To ensure that the TAG hydrolase activity resulted from mATGL288-Str and not from copurified enzymes, we introduced amino acid exchanges F17A and S47A to mATGL288-Str as control. TAG hydrolase activities are expected to be abolished because of exchange of a residue with a crucial role in substrate recruitment (F17) or the catalytic nucleophile (S47) (21, 28). mATGL288-Str and the mutant variants copurified with chaperones (Fig. 3*A*). mATGL288-Str exhibited higher activity than the protein expressed in Lemo21 (DE3) cells, showing activity in the basal state of 4000 nmol FA/(h ∗ mg protein) and an approximately threefold stimulation upon CGI-58 coactivation (Fig. 3*B*). The mATGL288-Str variants F17A or S47A did not exhibit TAG hydrolase activities. The fusion protein SMT3 served as another control and did neither lead to copurification of chaperons nor was it active in hydrolyzing TAG (Fig. 3*B*).

### Improved purification protocol of ATGL in ArcticExpress (DE3) cells provides highly active protein and enables chaperone removal

**Figure 4:**
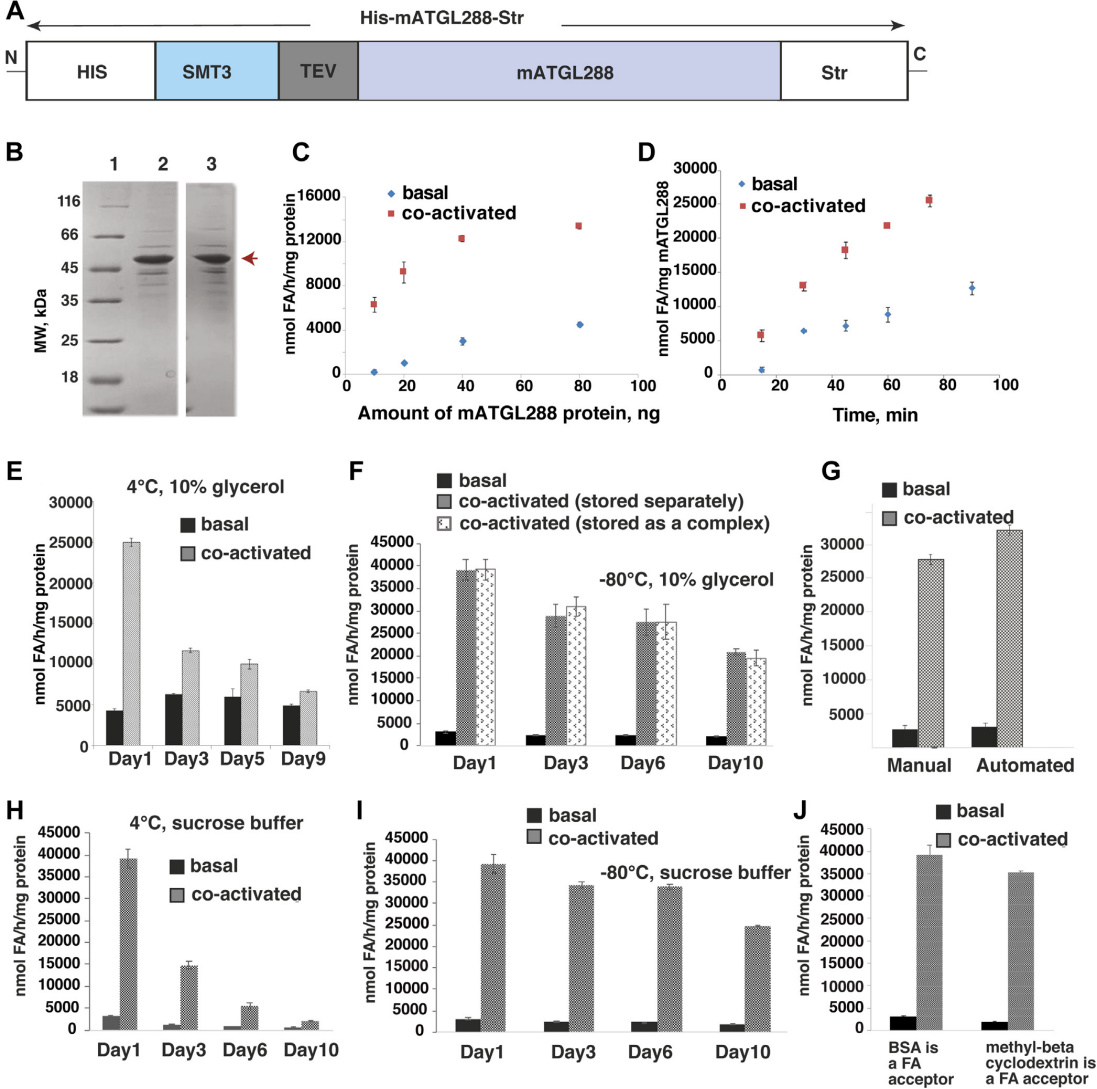
**Influence of improved purification, storage, and different FA acceptors on the activity of His-mATGL288-Str.***A*, schematic drawing of the double-tagged His-mATGL288-Str variant. *B*, comparison of manual and semiautomated purification of His-mATGL288-Str (46.1 kDa, *red arrow*). Lane 1, marker; lane 2, manual purification; lane 3, semiautomated purification. *C*, dose-dependence experiment of TAG hydrolytic activity in the presence and absence of the CGI-58 coactivator. *D*, time dependence of TAG activity in basal and CGI-58 coactivated conditions. *E*, changes in enzymatic activity in basal and CGI-58 coactivated conditions upon storage at 4 °C. *F*, basal and stimulated TAG hydrolytic activity after snap freezing (liquid nitrogen) and storage at −80 °C in 10% glycerol. His-mATGL288-Str was stored separately and as a His-mATGL288-Str/CGI-58 complex. *G*, TAG hydrolytic activity of purified His-mATGL288-Str after chaperone removal using manual and semiautomated purification. *H*, basal and stimulated TAG hydrolytic activity of His-mATGL288-Str and the His-mATGL288-Str/CGI-58 complex stored at 4 °C in sucrose buffer. *I*, basal and stimulated TAG hydrolytic activity of His-mATGL288-Str and the His-mATGL288-Str/CGI-58 complex after snap freezing (liquid nitrogen) and storage at −80 °C in sucrose buffer. Day 1 in panels *E*, *F*, *H*, and *I* refers to freshly purified protein. *J*, TAG hydrolytic activity of purified His-mATGL288-Str under basal and CGI-58 stimulated conditions in the presence of BSA and methyl-beta cyclodextrin as a fatty acid acceptor. BSA, bovine serum albumin; CGI-58, comparative gene identification-58; FA, fatty acid; Str, StrepII tag; TAG, triacylglycerol.

In order to improve the purification of mATGL288-Str while preserving the improved TAG hydrolase activity when expressed in ArcticExpress (DE3) cells (Fig. 3), we inserted an N-terminal His6 tag into the pST44-SMT3-TEV-mATGL288-STR plasmid (Fig. 4*A*) and termed the corresponding product His-mATGL288-Str. We supplemented the purification buffers for affinity purification with 10 mM MgCl_2_, 10 mM ATP, and high concentrations of KCl (100–500 mM, depending on the purification step). MgCl_2_ and ATP were added to aid in the removal of chaperones in further purification steps. Lane 2 in Figure 4*B* shows the improved chaperone removal compared with the purification shown in Figure 2. The purity of the proteins from both, the manual and the semiautomated purification protocol, was comparable (Fig. 4*B*, lanes 2 and 3). Yet, we observed an approximately threefold increase in yield. The increase from 0.4 mg protein to 1.1 mg purified protein in the semiautomated procedure most likely can be attributed to reduced handling and pipetting steps.

Time and dose-dependence experiments showed a linear increase in FA release of purified His-mATGL288-Str incubated for more than 75 min and at different amounts purified proteins in basal and coactivated conditions (Fig. 4, *C* and *D*).

Using freshly eluted proteins from the manual purification, TAG hydrolase activities were 2700 nmol FA/(h ∗ mg protein) in the basal state and 28,900 nmol FA/(h ∗ mg protein) in the CGI-58–stimulated state (Fig. 4*G*). The semiautomated purification resulted in comparable values (3100 nmol FA/[h ∗ mg protein]) for basal and CGI-58–stimulated (32,400 nmol FA/[h ∗ mg protein]) states (Fig. 4*G*). Remarkably, these data indicate a 10-fold increase in TAG hydrolase activity upon coactivation of freshly purified His-mATGL288-Str. Many enzymatic studies are carried out with stored or flash-frozen proteins. Therefore, we tested the influence of different storage conditions on enzymatic activity and coactivation. Samples stored at 4 °C prior the TAG hydrolase activity assays retained basal activity but showed a 50% loss in the potential to be coactivated by CGI-58 (Fig. 4*E*). Storage of purified His-mATGL288-Str at −80 °C resulted in almost no changes in basal TAG hydrolase activity (Fig. 4*F*). However, we observed substantial loss (ca. 50%) of activity upon coactivation within 10 days of storage at −80 °C (Fig. 4*F*). His-mATGL288-Str storage in sucrose buffer at 4 °C lead to marked loss of both: basal and coactivated activities (Fig. 4*H*). When His-mATGL288-Str alone and His-mATGL288-Str as complex with CGI-58 were frozen in sucrose buffer at −80 °C, we observed loss of activity upon coactivation by 40% (Fig. 4*I*). When we tested methyl-beta cyclodextrin as an FA acceptor in comparison to bovine serum albumin (BSA), we did not observe marked differences in TAG hydrolytic activities of His-mATGL288-Str in the basal and coactivated stages (Fig. 4*J*). It is worth noting here, that neither alpha-cyclodextrin nor beta-cyclodextrin served as FA acceptors for His-mATGL288-Str in our experiments (data not shown). In all assays, the coactivator CGI-58 was used from aliquoted samples stored at −80 °C as described in the Experimental procedures section, unless we stored the His-mATGL288-Str in complex with CGI-58 in the given buffer conditions.

### Purified mouse ATGL truncated after residue 288 exhibits transacylation activity

**Figure 5:**
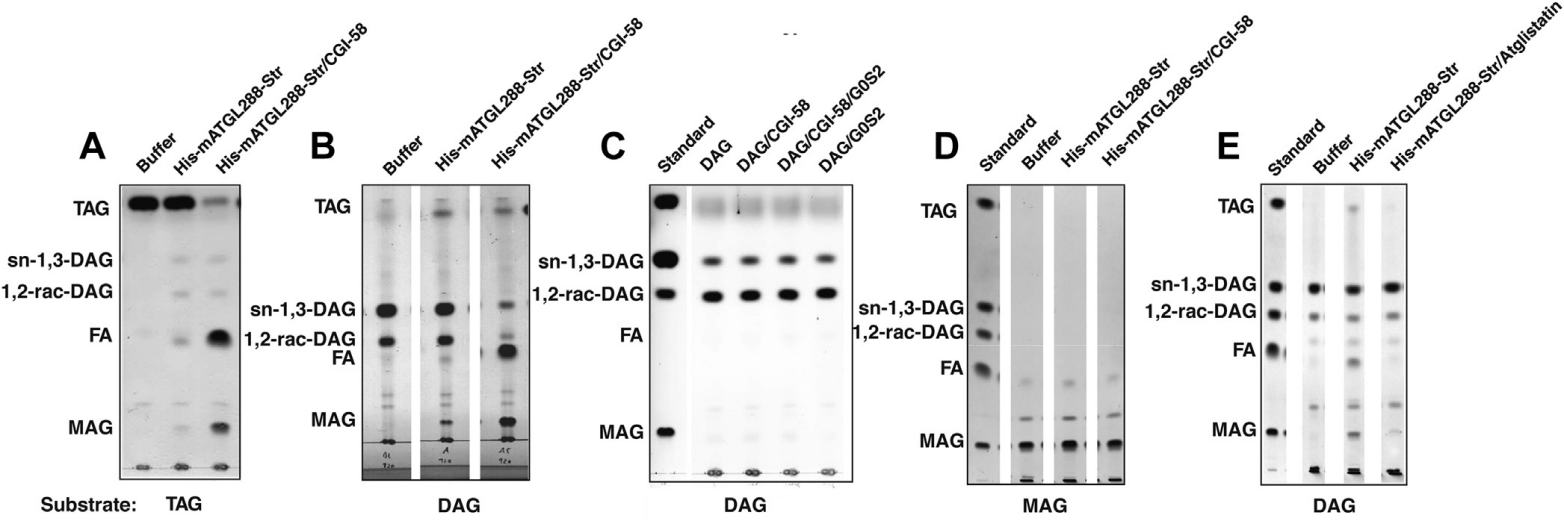
**Transacylase activity of His-mATGL288-Str.** TLC showing triacylglycerol build up with DAG as substrate indicates acyl-CoA independent transacylation. Basal and stimulated enzymatic activity of purified His-mATGL288-Str in the presence of (*A*) triacylglycerol (TAG) and (*B*) DAG as substrate. *C*, negative controls showing no enzymatic activities when DAG is offered as substrate to CGI-58 and G0S2. *D*, no enzymatic activity of His-mATGL288-Str is observed when monoacylglycerol (MAG) is offered as substrate. *E*, inhibitory effect of atglistatin on transacylation activity of His-mATGL288-Str. CGI-58, comparative gene identification-58; DAG, diacylglycerol; G0S2, G0/G1 switch gene 2.

Next, we analyzed the enzymatic products of purified His-mATGL288-Str in basal and coactivated stages *via* TLC. When using TAG as substrate under basal conditions, we observed an increase in lipid species corresponding to DAG, MAG, and FA and no depletion of TAG compared with the control (Fig. 5*A*). CGI-58–mediated coactivation of purified His-mATGL288-Str increased TAG hydrolysis and resulted in an accumulation of MAG and FA compared with the buffer control. We observed some signal corresponding to TAG and only marginal signal for DAG, indicating that the formation of DAG is not the end product of ATGL-mediated reaction (Fig. 5*A*). We did not observe self-hydrolysis of the TAG substrate using the buffer control. Residual TAG could stem from incomplete degradation of TAG and or a transacylation reaction, as suggested by earlier literature reports on ATGL activity (14, 15).

Therefore, we tested the transacylation activity of purified His-mATGL288-Str using DAG as substrate (Fig. 5, *B* and *C*). Under basal conditions, MAG and TAG increased. CGI-58 coactivation led to an even more pronounced increase in MAG and FA, a visible increase in TAG, and a marked decrease in DAG (Fig. 5*B*). Incubation of DAG with CGI-58 and/or G0S2 had no effect demonstrating that neither CGI-58 nor G0S2 exert enzymatic activity toward DAG (Fig. 5*C*). In addition, we analyzed the stereoselectivity/regioselectivity of transacylation by incubating ATGL in the absence or the presence of CGI-58 with either sn-1,3-DAG or 1,2-*rac*-DAG. In both cases, either using 1,2-*rac*-DAG or sn-1,3-DAG as substrates, DAG levels decreased and MAG levels increased. The effect could be seen especially when CGI-58–stimulated His-mATGL288-Str was used. In all cases tested, build up of TAG could be observed indicating that ATGL uses all DAG isomers tested for transacylation (Figs. S2 and S3).

We did not observe catalytic activity for His-mATGL288-Str or CGI-58–coactivated His-mATGL288-Str toward MAG (Fig. 5*D*). Atglistatin inhibited the transacylation activity (Fig. 5*E*). These observations indicate that ATGL/CGI-58 could perform coupled enzymatic reaction cycles of TAG hydrolysis and DAG transacylation in addition to possible DAG hydrolysis, which finally convert TAG to FA and MAG end products.

## Discussion

ATGL is one of the most important players in lipolysis. Yet, a detailed biochemical characterization is still limited because of experimental difficulties in overexpression and purification of the protein. In this study, we present methods and protocols that allow the expression and purification of active ATGL starting with the selection of expression hosts. Choosing the right expression host for protein expression is the key for success. Protein folding, solubility, activity, stability during purification, and the final yield are often crucial factors for protein production. Bacterial expression hosts are frequently used because they offer a robust and cost-effective expression system that typically provide high protein yields in the shortest amount of time. Different *E. coli* strains are engineered and genetically well characterized, which enables further fine-tuning of overexpression conditions. Furthermore, the scale up in an academic laboratory setting is usually easily implemented. Mammalian expression systems are capable of producing proteins with post-translational modifications more closely resembling those of humans. However, they usually provide lower yields and are more demanding and expensive in their handling. Alternatively, insect cell expression systems have often been successfully used for expression of eukaryotic proteins and multiprotein complexes (29). In this article, we show a large-scale approach for expressing ATGL in *E. coli*, mammalian cells (HEK), and insect cells (Sf9) at the OPPF-UK. Based on these results, we optimized protocols for bacterial overexpression and purification for an SMT3-tagged ATGL variant truncated after D288. *E. coli* Lemo21 (DE3) cells are used by default at OPPF-UK because they allow tunable expression of difficult targets like membrane proteins, toxic proteins, and proteins prone to insoluble expression. In many cases, less expression equals more protein of interest produced in the desired form. Even in the native cellular environment, protein folding is not always a spontaneous event. The protein synthesis machinery may produce aberrantly folded proteins prone to aggregation or rapid intracellular degradation. Therefore, cells invest in a complex network of molecular chaperones, which provide constant surveillance for protein homeostasis; owing to the great capacity of these molecular machines, the GroE chaperonin system is successfully employed in protein refolding strategies and biotechnological applications (30, 31).

Our results show that full-length ATGL and many tested constructs are difficult to express in a soluble form from various host systems (Fig. 1*B*). We have achieved reasonable expression levels of ATGL in a very active form when expressing and purifying ATGL N-terminally fused to an SMT3 tag and truncated after amino acid D288 (Figs. 1*B* and 2). Based on previous reports, we are confident that major catalytic activities and protein–protein interaction modules are present within this ATGL variant, allowing extrapolation of the observed activities also to full-length ATGL. The recombinant protein copurified with DnaK and GroEL, which belong to *E. coli* chaperonins, facilitating protein folding. Typically, these chaperones lose their activity at low temperatures. For example, the *E. coli* chaperonin complex only maintains 30% refolding activity at 12 °C compared with its activity at the temperature optimum of 30 °C. In order to avoid protein misfolding and achieve better expression of ATGL, we changed the expression host to ArcticExpress (DE3) cells, which were designed for improved protein processing and high protein refolding activities even at low temperatures (4–12 °C) (32). In our purification optimization, we introduced an additional N-terminal purification tag and also drew on literature reports regarding chaperone function. DnaK is influenced by potassium ions *via* stabilization of a DnaK–ATP complex. Two K^+^ and one Mg^2+^ are bound in the ATPase site essential for the ATP-induced binding and release of peptide ligands (33). As a side effect, high KCl concentrations increase the binding specificity of recombinant His6-tagged proteins during purification. GroEL is also stabilized by divalent cations and shows the highest activities in the presence of magnesium (34). The binding and release of substrate protein in the GroE chaperonin complex is also highly ATP dependent. The introduction of two different affinity tags (His6 and Strep), together with the presence of ATP, Mg^2+^, K^+^, and glycerol in our purification protocol enabled removal of majority of the chaperonins, while achieving highest reported enzymatic activities against TAG of recombinantly expressed and purified ATGL in basal and CGI-58–stimulated states.

In addition to the increase in overall yield in the semiautomated procedure, the purification scheme uses less disposal material (*e.g.*, collection tubes), which in addition to economic and ecological savings, also might reduce loss of His-mATGL288-Str because of unspecific binding.

TAG hydrolytic activity of ATGL has been shown from laboratories worldwide. However, the current literature refers mostly to cellular lysates containing ATGL. The measured activity of full-length ATGL in cell lysates is in the nanomolar range per hour and milligram protein (14, 20, 25, 35, 36, 37). We demonstrate that high TAG hydrolase activity of the ATGL variant truncated after residue D288 in both, basal and CGI-58–stimulated form, with an FA release in the micromolar range per hour and milligram protein. The presented expression and purification protocols open the route for in-depth biochemical, structural, and kinetic characterizations of ATGL in future studies.

Fresh protein gives the best results in terms of activity and the capacity to be coactivated by CGI-58. While basal activities are almost not affected upon storage or freezing, drastic losses in coactivation are observed. The loss of activation activity could be explained by conformational changes of ATGL upon storage or freezing, which seems to affect the CGI-58 interaction site. In this regard, it is exceptionally important to use the freshly purified protein for the assays, especially when quantitative studies of ATGL activities are being performed.

A few reports also demonstrated transacylation activity of ATGL in mammalian tissue (15) and in *in vitro* studies of ATGL expressed in Sf9 cells (14). The reported numbers of transacylation activity of ATGL are in the range of 0.4 nmol per minute and milligram protein for Sf9 cells (14). It should be noted here, that different from our purified and shortened variant of mouse ATGL expressed in *E. coli*, Jenkins *et al.* had used affinity-purified full-length human ATGL produced in insect cells. A recent study demonstrated transacylation activity of ATGL *in vivo* using white adipose tissue of mice with measured transacylation activity in the range of ∼0.3 to 0.9 nmol per hour and milligram protein after 5 and 24 h of fasting, respectively, and in the range of 2 to 5 nmol per hour and milligram protein in LD extracts (15). It is well established that TAG synthesis is not the mere reverse reaction of TAG hydrolysis since unesterified FAs need to be activated with coenzyme A generating acyl-CoAs. MAG and DAG transacylases do not require acyl-CoA donors and may confer DGAT-independent TAG synthesis in some tissues like the intestine, but their mechanisms are not fully understood (38, 39, 40). Our *in vitro* experiments with purified ATGL variant revealed transacylation activity in the absence of acyl-CoA synthetase, coenzyme A, and cofactors (ATP) necessary for FA activation. Accordingly, our results reveal that the observed DAG transacylase activity is indeed an intrinsic property of ATGL. In view of these different reactions at the water–lipid interphase, we refrained from computing apparent kinetic parameters since key concepts of neither Michaelis–Menten kinetics nor an allosteric sigmoidal kinetics are likely not to be fulfilled. In agreement with the data shown by Zhang *et al.* (15), the transacylation activity can be inhibited by atglistatin, a synthetic competitive inhibitor optimized for inhibiting the hydrolytic activity of mouse ATGL. The causes for the atglistatin-mediated inhibition could be that both activities employ the same catalytic center or that hydrolysis directly precedes the transacylation reaction. In the presence of DAG as a sole substrate, ATGL mediates the disproportionation of DAG into TAG and MAG and very likely also the degradation of DAG into MAG and FA. The activity of ATGL toward DAG is greatly enhanced in the presence of CGI-58. However, whereas large amounts of MAG and FA are formed under these conditions, the generation of TAG remains in a steady state. This could suggest that ATGL/CGI-58 catalyzes cycles of DAG transacylation and TAG hydrolysis ultimately leading to the formation of MAG and FA. At the current stage, it is not clear, what percentage of MAG and FA is generated *via* a direct hydrolytic reaction (DAG to MAG and FA) or *via* transacylation (two DAG molecules to TAG and MAG, followed by hydrolysis of TAG to DAG and FA).

Analysis of the positional selectivity of ATGL using mass spectrometry had revealed that ATGL predominantly cleaves the sn-2 position of TAG, consequently generating sn-1,3 DAG. Upon coactivation with CGI-58, increased activity on sn-1 or sn-3 esters had been observed (41). That study had only investigated the substrate and stereoselectivity of ATGL by characterization of the produced DAG isoforms. MAG as potential outcome of the reaction with ATGL had not been investigated, since the activity of ATGL on DAG had not been in the spotlight at that time. However, the authors reported barely detectable levels of the sn-1,2 or sn-2,3 DAG levels, which is in line with a fast turnover (either *via* transacylation or subsequent hydrolysis) of DAGs. It also has to be kept in mind, that the TAG degradation products DAG and MAG might be rapidly used as precursors for neutral and glycerophospholipid synthesis or signaling molecules under *in vivo* conditions. Using our experimental setup, we did not observe any transacylation or TAG build up using MAG as substrate, which differs from a previous report (14). One possible explanation for the differences could result from different experimental setups. We offered only one type of substrate (DAG or MAG), whereas previous studies used mixture of monoolein as donor and diolein. We speculate that when monoolein and diolein are mixed together, it opens the routes for ATGL to form another TAG, which might explain the build up of TAGs observed by Jenkins *et al.* (14). Another explanation could be differences in water–liquid interface: According to the literature, the amount of water in a reaction mixture can determine the direction of lipase-catalyzed reaction: When there is water deficiency, only esterification and transesterification reactions are favored, whereas hydrolysis is the preferred reaction in excess of water (42). We speculate that ATGL-mediated TAG hydrolysis and DAG transacylation reactions are coupled. Because of this, the DAG transacylase activity aids in TAG degradation rather than TAG synthesis. Zhang *et al.* (15) showed that ATGL acts as transacylase in adipose tissue in the absence of HSL. Such a transacylation activity of ATGL could also explain the phenomenon of lipolysis in HSL-deficient patients and mice, where only ATGL performs initial vital lipolytic reactions steps (43, 44, 45). The detailed reaction mechanisms for possible ATGL reactions, namely hydrolysis of TAG to DAG to MAG and/or hydrolysis of TAG to DAG followed by transacylation of DAG to yield TAG and MAG, are still unknown and await further, intensive, investigation. It is also possible that both mechanisms take place in parallel or take place at different levels under different physiological conditions in different tissues. In support of this possibility are recent experiments showing some hydrolytic activity of ATGL on sn-1,3 and sn-2 FA esters of hydroxy FAs—glycerol esters and that ATGL exhibits transacylase activity in the remodeling of TAG estolides, yet these appear to be poorer substrates than normal FA esters (46). Obviously, these findings add to the complexity of possible reactions catalyzed by ATGL.

Taken together, the available data support the notion that hydrolytic and transacylation activities of the intracellular lipases ATGL and HSL are overlapping and coupled, yet the physiological significance is still incompletely understood (12, 15, 47). Apparently, ATGL can use DAG as both, acyl acceptor and acyl donor, and thus resynthesizes TAG and generates MAG during the reaction. Our study corroborates the emerging notion that PNPLA enzymes are multifunctional enzymes and that the processes of TAG mobilization might not strictly sequential as assumed in most current models of lipolysis (48).

With the possibility to produce active ATGL with protocols presented here, important ground is laid for detailed biochemical and enzymatic characterization of ATGL in controlled environments.

## Experimental procedures

### Materials

Trioleoylglycerol, dioleoylglycerol (mixture of 65% of 1,3-sn-DAG and 35% of 1,2-*rac*-DAG), or monoolein (1-*rac*-monooleoyl glycerol), other chemicals, antibiotics, and buffers were obtained from Sigma–Aldrich or Carl Roth GmbH if not stated otherwise. Columns for protein purification were obtained from Cytiva (formerly GE Healthcare Life Sciences). [9,10-^3^H]-Triolein was obtained from PerkinElmer Life Sciences. BSA was ordered from Thermo Fisher Scientific.

### ATGL truncations and high-throughput cloning of ATGL at OPPF-UK

As described in the Results section, we combined structure and domain predictions with experimental data of mouse ATGL (486 amino acids) and human ATGL (504 amino acids). These factors resulted in different variants of mouse ATGL, namely full-length mATGL, M1-Q257, M1-E272, M1-D288, M1-K295, M1-D364, and M1-H390 (Fig. 1*A*). The cloned constructs code for just a C-terminal StrepII tag or for an N-terminal His6 tag combined with five variable fusion protein tags (GST, MBP, NusA, SMT3, and TRX) located N-terminally of the respective ATGL variants (Table 1). Using primers listed in Table S1, we amplified the coding regions with Phusion Flash II DNA Polymerase (Thermo Fisher Scientific) or KOD Xtreme Hot Start DNA Polymerase (Novagen) in 96-well PCR plates employing 60 °C as annealing temperature for all reactions. Amplified PCR products were subjected to a single purification step with the AMPUre XP PCR (Beckman Coulter) kit, methylated template DNA digested with DpnI, and cloned into the pOPIN vector suites, which is based on the pTriEX vectors from Merck enabling overexpression in different heterologous hosts (49), and transformed into OminMAX 2 cells (Thermo Fisher Scientific) using in-fusion technology combined with blue–white screening detected by X-Gal.

### mATGL production and solubility screening in different hosts at OPPF

All selected vectors of the OPIN suite allowed protein expression in multiple hosts systems like *E. coli* (Rosetta 2 (DE3); Lemo21 (DE3)), mammalian cell lines (HEK293T), and insect cells (*Sf9*). Small-scale test expressions were carried out in 24-well plates. Transformed *E. coli* cells for manual induction were grown in power broth supplemented with appropriate antibiotics at 37 °C. Overexpression was induced upon addition of 0.1 mM IPTG for Lemo21 (DE3) and Rosetta 2 (DE3) cells followed by overnight incubation at 20 °C. Transformed *E. coli* strains for autoinduction were grown in Express Instant TB Medium (Novagen) at 25 °C for 24 h (50). Cells were harvested by centrifugation, and the growth media were decanted. Upon freezing and thawing, the cells were lyzed in buffer containing 50 mM NaH_2_PO_4_, 30 mM NaCl, 10 mM imidazole, 1% (v/v) Tween-20, pH 8.0 supplemented with 1 mg/ml lysozyme (Merck) and 3 units/ml benzonase (Merck). The lysates were cleared by centrifugation of the deep-well plates and transferred to microtiter plates containing a suspension of magnetic nickel (II) ion–nitrilotriacetic acid (Ni^2+^–NTA) beads (Qiagen). Using a 96-well magnet (Qiagen) allowed the removal and wash (50 mM NaH_2_PO_4_, 300 mM NaCl, 20 mM imidazole, 0.05% [v/v] Tween-20, adjusted to pH 8.0) of protein, which was not binding to the Ni–NTA beads. Bound protein was eluted from the beads in buffer containing 50 mM NaH_2_PO_4_, 300 mM NaCl, 250 mM imidazole, 0.05% (v/v) Tween-20, adjusted to pH 8.0, and transferred to fresh plates. Individual protein expression levels were analyzed using 26-well SDS-PAGE gels and by manual inspection of bands at the expected molecular weights of the different fusion proteins. Pellet fractions were solubilized in 500 μl of resuspension buffer (50 mM Tris, pH 8.5, 300 mM NaCl, 8 M urea, 30 mM imidazole, 0.5% [v/v] Triton X-100, 1 mM Tris(2-carboxyethyl)phosphine [TCEP]) for further expression screening by denaturing conditions in 96-well plates using affinity chromatography and Ni^2+^–NTA Sepharose. Bound protein was eluted with in the same buffer containing 300 mM imidazole and analyzed using SDS-PAGE.

### Expression screening of mATGL in mammalian cell lines (HEK293T)

Resulting pOPIN vectors harboring the genes for mATGL variants described here were tested for soluble protein expression according to OPPF protocols (https://www.oppf.rc-harwell.ac.uk/OPPF/protocols/sop/OPPF-UK%20SOP%20Mammalian%20Expression%2020161010.pdf). Briefly, HEK293T cells grown attached in 24-well plates in Dulbecco's modified Eagle's medium with 2% fetal calf serum were transfected with a cocktail containing plasmid DNA, serum-free Dulbecco's modified Eagle's medium, and GeneJuice (Merck) as transfection agent. The HEK293T cells express the SV40 large T-antigen allowing episomal amplification of the plasmids. The transfected cells were incubated at 37 °C in a humidified 5% CO_2_ environment for 3 days and then washed with PBS buffer. Cells were lysed in buffer containing 50 mM NaH_2_PO_4_, 30 mM NaCl, 10 mM imidazole, 1% (v/v) Tween-20, and pH 8.0 supplemented with COMPLETE protease inhibitors (Roche) and 3 units/ml benzonase (Merck). The lysates were centrifuged at 12,000*g* for 10 min, and the expression of ATGL variants in the solubilized fraction assessed by Western blotting using an anti-His antibody (AntiHis, R&D Systems; MAB050) following the manufacturer's instruction.

### Expression screening of mATGL in the insect cell line Sf9

Resulting pOPIN transfer vector harboring genes for mATGL variants (described herein) were cotransfected with a genetically disabled baculovirus propagated as a bacmid according to OPPF protocols (https://www.oppf.rc-harwell.ac.uk/OPPF/protocols/sop/OPPF-UK%20SOP%20Insect%20Expression%2020161006.pdf). In brief, Sf9 cells were transfected with bacmid using FuGene HD Transfection Reagent in 24-well culture plates and incubated for 6 to 7 days at 27 °C to generate a primary virus P_0_ stock from the supernatant, which was then used to generate a P1 virus stock. Target protein expression was tested in suspension using P1 virus in different dilutions in 24-deep well blocks. Expression was performed at 27 °C for 3 days followed by robotic Ni^2+^–NTA expression screen for soluble protein using lysis buffer containing 1% (v/v) Tween-20 as described previously for *E. coli* expression testing.

### Cloning of ATGL, CGI-58, and G0S2

The coding sequence for mATGL was ordered as an *E. coli* codon-optimized gene (Eurofins Genomics), the coding sequence of SMT3 was amplified based on a previously described construct (51), and the glycine linker together with the tobacco etch virus (TEV) cleavage site were included *via* the primer sequences (Table S2). The PCR was carried out with the Q5 High-Fidelity DNA Polymerase (NEB) according to the manufacturer's protocol. The pST50-Trc4-DHFRSTR donor vector was linearized using the BamHI and NgoMIV restriction sites (52). We used agarose gel electrophoresis to verify the correct fragment length of the linearized pST50-Trc4 vector and the PCR-derived fragment (the insert coding for an SMT3 fusion protein, followed by a glycine linker and the coding sequences for a TEV protease cleavage site as well as for mATGL). Vector and insert were mixed following the Gibson Assembly protocol (NEB) and transformed into NEB 5-alpha competent *E. coli* cells according to the manufacturer's protocols. We then used restriction-free cloning to transfer this sequence into the pST44 vector. For that purpose, we linearized the pST44 polycistronic plasmid by a double digest using restriction enzymes BspEI and MIuI specific for the desired translational cassette 4 and inserted the sequence from the pST50 donor vector. Because of better expression levels and solubility, the pST44 construct of mATGL was truncated at amino acid D288 (Table 2). Primers used for the implementation of the amino acid exchanges F17A and S47A are also listed in Table 2. To improve the purification of mATGL truncation M1—D288, an N-terminal tag was introduced using the Q5 Site-Directed Mutagenesis Kit (NEB). Furthermore, a codon-optimized sequence for human G0S2 was transferred into translational cassette 1 of a pST44 vector by restriction-free cloning using the Gibson Assembly Cloning Kit, generating the expression plasmid pST44-SMT3-TEV-hG0S2-HIS. In a similar manner, a synthetic codon-optimized sequence was inserted into the pST44 translational cassette 3 for overexpression of an SMT3-tagged version of mouse CGI-58 (pST44-SMT3-TEV-mCGI58-HIS).

**Table 2:** Primers used for this work

Variant	Forward primer	Reverse primer	Vector
M1-D288 (mouse ATGL truncation)	AAGGAGATATACATATGGGATCCATGTCGGACTCAGAAGTCAATC	CCGCCACCACCAATCTGTTCTCTGTGAGC	pST50
M1-D288 (mouse ATGL truncation)	TTTTCCCAGTCGAGCTCAATAATTTTGTTTAACTTTAAGAAGGAGA TATAC	TGTTATCCGCTGGTACCTGTACATTAGTGGTGGTGGG	pST44
N-terminal His insertion	CACCATCACTCGGACTCAGAAGTCAATC	ATGGTGATGCATGGATCCCATATG TATATC	pST44
F17A of mATGL288-Str	AGGCTGTGGCGCGCTGGGCGTTT	GCAAAACTGATGTTCCATTTGG	pST44
S47A of mATGL288-Str	TTATGGTGCGGCGGCAGGCGCCC	ATGTGCGTAGCGTTGGCCAC	pST44
SMT3	GCCGGCAGCGCTTGGAGC	GCCCTGAAAATACAGGTTTTCTCCGCC	pST44

The sequences of all generated inserts were verified by DNA sequencing (Microsynth). For reasons of clarity and readability, we call the expression product of the expression plasmid pST44-SMT3-TEV-mATGL288-STR: “mATGL288-Str” and that of pST44-HIS-SMT3-TEV-mATGL288-STR: “His-mATGL288-Str.” The proteins expressed from the plasmids pST44-SMT3-TEV-hG0S2-HIS and pST44-SMT3-TEV-mCGI58-HIS are referred to as “G0S2” and “CGI-58,” respectively, throughout the article. As shown in different previous articles, N-terminal protein tags do not influence the inhibitory and stimulatory effect of G0S2 and CGI-58, respectively (10, 25, 51).

### Expression of mATGL288-Str in Lemo21 (DE3) cells

The expression plasmid pST44-SMT3-TEV-mATGL288-STR was transformed into chemically competent *E. coli* Lemo21 (DE3) cells. After plating on selection medium containing 35 μg/ml chloramphenicol, 100 μg/ml ampicillin, and 2% glucose, individual colonies were picked to inoculate 15-ml day cultures containing the same antibiotics. To suppress leaky expression, the LB medium was enriched with 2% glucose. Eight hours later, 5 ml of overnight cultures (ONCs) were used to inoculate 250-ml ONCs. These were transferred on the next morning into baffled flasks with 2750-ml LB medium with no further antibiotics but 0.5% glucose and incubated at 37 °C. In addition, 300 μl of silicone oil were added to each flask to reduce foam formation while shaking at 120 rpm. The cultures were cooled to 16 °C and at an absorbance of 0.5 at 600 nm. Recombinant protein expression was induced by 0.1 mM IPTG (Roth). After overnight induction, the cells were harvested by centrifugation at 6000*g* for 20 min at 4 °C. Until further usage, the cell pellets were frozen and stored at −20 °C. The achieved protein expression levels were analyzed using SDS-PAGE.

### Expression of His-mATGL288-Str in ArcticExpress (DE3) cells

The expression of pST44-HIS-SMT3-TEV-mATGL288-STR plasmid was transformed into the chemically competent ArcticExpress (DE3) cells and plated on LB agar plates containing 100 μg/ml ampicillin followed by overnight incubation at 37 °C. The next day, 15-ml LB medium supplemented with 100 μg/ml ampicillin, 20 μg/ml gentamycin, and 2% glucose was inoculated with a single colony and grown for 8 h at 37 °C (day culture). Then, 250-ml LB medium with the same supplements was inoculated with 5 ml of the day culture and cultured overnight at the same conditions (ONC). The following day, 5-l baffled flasks containing 2750 ml of LB medium supplemented with 0.5% glucose, 300 μl of silicone oil, and 10 mM MgCl_2_ to support chaperone removal during protein isolation were inoculated with the total volume of ONC. No selection antibiotics were used for the expression cultures. While shaking with 120 rpm at 30 °C, the cells were grown until an absorbance of 0.5 at 600 nm, before induction with 0.1 mM IPTG at 10 °C for 24 h. Finally, the cells were harvested by centrifugation at 6000*g* and 8 °C, and the resulting cell pellet was frozen until further usage at −20 °C. The expression of mATGL288-Str variants F17A und S47A in ArcticExpress (DE3) cells followed the same protocol.

### Purification of mATGL288-Str expressed in Lemo21 (DE3) cells

Frozen Lemo21 (DE3) cell pellets with the overexpressed protein were thawed on ice and resuspended in lysis buffer A (100 mM K_2_HPO_4_/KH_2_PO_4_ pH 7.5, 150 mM KCl, 10% [v/v] glycerol, 0.1% [v/v] IGEPAL CA-630, and 1 mM TCEP) supplemented with 1.5 nM pepstatin A, 3.3 nM antipain, and 43 nM leupeptin as protease inhibitors. The cell pellet derived from a 1 l culture was resuspended in 100 ml of lysis buffer A. The cells were disrupted by sonication on ice using the SONOPLUS ultrasonic homogenizer HD 2070 (BANDELIN) in combination with an RZ3 processing vessel (BANDELIN). After centrifugation at 40,000*g* for 60 min on 4 °C, the lysate was cleared by filtration using a 1 to 2 μm glass fiber prefilter in combination with 0.45 μm regenerated cellulose syringe filters. Subsequently, the lysate was loaded onto a 5 ml StrepTrap HP column using an ÄKTA Avant chromatography system (Cytiva, formerly GE Healthcare Life Sciences). To reduce the concentration of IGEPAL CA-630 below the critical micelle concentration, the column was washed with ten column volumes (CVs) of wash buffer A (100 mM K_2_HPO_4_/KH_2_PO_4_, pH 7.5, 150 mM KCl, 10% [v/v] glycerol, 0.003% [v/v] IGEPAL CA-630, and 1 mM TCEP). The UV absorbance was set to zero at the end of the washing to enable peak detection after elution. The soluble and bound target protein mATGL288-Str was eluted in ten CVs of a 0 to 100% gradient of elution buffer A (100 mM K_2_HPO_4_/KH_2_PO_4_, pH 7.5, 150 mM KCl, 10% [v/v] glycerol, 0.003% [v/v] IGEPAL CA-630, 1 mM TCEP, and 2.5 mM desthiobiotin). The recombinant protein eluted at approximately 60% of elution buffer A, and the collected fractions were pooled according to purity after analysis on SDS-PAGE. After a concentrating step using Centricon tubes with 30-kDa cut off, the sample was subjected to SEC using a HiLoad 16/60 Superdex 200 pg column in 100 mM K_2_HPO_4_/KH_2_PO_4_, pH 7.5, 150 mM KCl, 10% (v/v) glycerol, 0.003% (v/v) IGEPAL CA-630, 1 mM TCEP, and 1 mM EDTA. Fractions containing mATGL288-Str were pooled and analyzed by SDS-PAGE. The mATGL288-Str variants F17A und S47A expressed in ArcticExpress (DE3) cells were purified using the same protocols. Unless stated otherwise, TAG hydrolase activity assays were carried out directly after purification.

### Purification of the His-mATGL288-Str variant

Purification of the His-mATGL288-Str variant was performed *via* sequential affinity chromatography utilizing first the His tag and second the StrepII tag. In the first purification trials, the protein was first bound to and eluted from a His-affinity matrix. Then, the pooled fractions were bound to and eluted from a Strep column. To facilitate an automated purification using HisTrap HP and StrepTrap HP columns, the ÄKTA avant 25 system was upgraded with an additional versatile valve (V9-V) between the injection valve (V9-Inj) and the column valve (V9-C). This modification offered the possibility to selectively add or remove chromatography columns within the current flow path during certain purification steps. For the preparation of crude cell extracts, frozen ArcticExpress (DE3) cell pellets (from 1 l culture) with the overexpressed target protein were thawed on ice and resuspended in 25 ml lysis buffer B (100 mM K_2_HPO_4_/KH_2_PO_4_, pH 7.5, 100 mM KCl, 10% [v/v] glycerol, 0.1% [v/v] IGEPAL CA-630, 30 mM imidazole, 1 mM TCEP, 10 mM ATP, and 10 mM MgCl_2_) supplemented with 1.5 nM pepstatin A, 3.3 nM antipain, and 43 nM leupeptin as protease inhibitors. The *E. coli* cells were disrupted by sonication on ice using the SONOPLUS ultrasonic homogenizer HD 2070. After centrifugation at 40,000*g* for 60 min on 4 °C, the lysate was cleared by filtration using a 1 to 2 μm glass fiber prefilter in combination with 0.45 μm RC syringe filters. First, 1 ml HisTrap HP and StrepTrap HP columns were both equilibrated using 12 CVs of lysis buffer B or equilibration buffer B (100 mM K_2_HPO_4_/KH_2_PO_4_, pH 7.5, 100 mM KCl, 10% [v/v] glycerol, 1 mM TCEP), respectively. Loading of the sample onto the HisTrap HP column was followed by washing steps using five CVs of lysis buffer B, seven CVs of Ni^2+^–wash buffer B (100 mM K_2_HPO_4_/KH_2_PO_4_, pH 7.5, 500 mM KCl, 10% [v/v] glycerol, 30 mM imidazole, 1 mM TCEP, 0.003% [v/v] IGEPAL CA-630, 10 mM ATP, and 10 mM MgCl_2_), and ten CVs of equilibration buffer B. The latter followed the purpose to remove remaining concentrations of ATP showing a high UV signal. Nonspecific protein bound to the immobilized metal ion affinity chromatography resin was removed by a step gradient of 20% Ni^2+^–elution buffer B (100 mM K_2_HPO_4_/KH_2_PO_4_, pH 7.5, 100 mM KCl, 10% [v/v] glycerol, 1 mM TCEP, 0.003% [v/v] IGEPAL CA-630, and 500 mM imidazole) over 15 CVs, followed by a reset of the UV absorbance to zero. Prior to elution from the HisTrap HP column, the versatile valve was used to switch the StrepTrap HP column into the flow path. Subsequently, His-mATGL288-Str was eluted directly onto the StrepTrap HP column using a 100% step gradient of Ni^2+^–elution buffer B. Afterward, the column valve was switched to “bypass” to exclude the HisTrap HP column from the current flow path. Washing with ten CVs of equilibration buffer B completely removed leftover IGEPAL CA-630 and imidazole concentrations. Finally, the recombinant protein eluted in ten CVs of strep-elution buffer B (100 mM K_2_HPO_4_/KH_2_PO_4_, pH 7.5, 100 mM KCl, 10% [v/v] glycerol, 1 mM TCEP, and 10 mM desthiobiotin). The purified protein was further analyzed using SDS-PAGE. Unless stated otherwise, TAG hydrolase activity assays were carried out directly after purification or upon protein snap freezing in liquid nitrogen to check the influence of freezing on the hydrolytic activity.

### Expression and purification of G0S2 and CGI-58

Human G0S2 and mouse CGI-58 were expressed from the plasmids pST44-SMT3-TEV-hG0S2-HIS and pST44-SMT3-TEV-mCGI58-HIS in ArcticExpress (DE3) cells as described for His-mATGL288-Str, without the additional pre-day culture. Cell pellets with overexpressed G0S2 or CGI-58 were lysed by sonication in lysis buffer C (20 mM Tris–HCl, pH 7.8, 300 mM NaCl, 30 mM imidazole, 0.1% [v/v] IGEPAL CA-630, and 1 mM TCEP) supplemented with 1 mM benzamidine and 0.1 mM PMSF used as protease inhibitors. Soluble fractions of G0S2 and CGI-58 were loaded onto 5 ml HisTrap HP columns using a 50-ml superloop. An additional washing step with ten CVs of wash buffer C (20 mM Tris–HCl, pH 7.8, 300 mM NaCl, 30 mM imidazole, 10% [v/v] glycerol, and 1 mM TCEP) was performed to remove remaining concentrations of IGEPAL CA-630 to avoid interference with activity assays. The proteins were eluted in a gradient of elution buffer C (20 mM Tris–HCl, pH 7.8, 300 mM NaCl, 250 mM imidazole, 10% [v/v] glycerol, and 1 mM TCEP). Eluted fractions were analyzed by SDS-PAGE. After determination of the protein concentration using the Bradford method, the samples were snap frozen in liquid nitrogen and stored at −80 °C until further usage.

### Cloning, expression, and purification of SMT3

As additional control for unspecific TAG hydrolase activity, a construct only including SMT3 was cloned by deleting the coding sequence for mATGL288 from the pST44-SMT3-TEV-mATGL288-STR plasmid (Table 2). Expression and purification of SMT3 protein was carried out as described for the ATGL variants.

### TAG hydrolase activity assay

TAG hydrolase activity assays were performed essentially as described (20), yet on a smaller scale. The TAG substrate consisted of 1 mM triolein containing 11.25 μCi/ml [9,10(N)-^3^H]-glycerol trioleate as a tracer and 109 μM of a 3:1 phosphatidylcholine/phosphatidylserine mixture in 0.1 M potassium phosphate buffer at pH 7.0. Micelles were prepared on ice by sonication using the Virsonic 475 ultrasonic cell disruptor (Virtis Virsonic) with 20% of output power for 1 min with intervening breaks of similar length. Based on the performed enzymatic experiments, the substrate was diluted to the desired substrate concentrations ranging from 0.02 to 1 mM. In addition, each dilution was supplemented with FA-free BSA (Sigma–Aldrich) to reach a final concentration of 5% (w/v). When enzymatic activity of ATGL288 was tested in the presence of methyl-beta cyclodextrin, the last was used to reach the final concentration of 7.5 g/l. To determine the TAG hydrolase activity of mATGL288, 40 ng of purified protein in a total volume of 25 μl sucrose solution (250 mM sucrose, 1 mM EDTA, pH 7.0, and 1 mM DTT) were incubated with the equal volume of substrate solution to get 50 μl of the final reaction mixture. In case of the basal enzymatic activity, the individual reactions were incubated in a water bath at 37 °C for 60 min. To measure stimulated activity, reactions were incubated for 30 min with equimolar amounts of mCGI-58. About 25 μg of total protein in a cell lysate were included as positive control and treated identically. As a blank measurement, 25 μl of sucrose solution were mixed with 25 μl of substrate solution (50 μl of reaction mixture). The enzymatic reactions were terminated by the addition of 650 μl of methanol/chloroform/*n*-heptane (10:9:7) and 200 μl of 0.1 M potassium carbonate (pH of 10.5). FA extraction was performed by mixing the samples vigorously for 5 s followed by centrifugation at 500*g* and room temperature for 10 min. An aliquot of 200 μl of the upper aqueous phase was transferred into scintillation vials containing 2 ml of scintillation cocktail (Roth). Radioactivity was determined by liquid scintillation counting using the LS 6500 Multipurpose Scintillation Counter (Beckman). Specific substrate activities were in the range of 1000 to 2000 cpm/nmol. Counts generated from blank measurements were subtracted and the rates of TAG hydrolase activity, presented as nanomoles of released FA per hour and milligram of protein, were calculated according to Schweiger *et al.* (20).

To examine the storage conditions of His-mATGL288-Str at different time points, we performed TAG hydrolase activity assays, using the assay described previously with the only difference that the samples were either stored at 4 °C or at −80 °C (after flash freezing) prior to the assays at later time points. The samples were stored in two ways: (1) in strep-elution buffer B (100 mM K_2_HPO_4_/KH_2_PO_4_, pH 7.5, 100 mM KCl, 10% [v/v] glycerol, 1 mM TCEP, and 10 mM desthiobiotin) and (2) in sucrose buffer (250 mM sucrose, 1 mM EDTA, pH 7.0, and 1 mM DTT). We have also tested whether His-mATGL288-Str alone and stored together as complex with CGI-58 makes a difference in sample storage. In order to do so, we stored His-mATGL288-Str alone and as mATGL288-CGI-58 complex in strep-elution buffer B at −80 °C.

### TLC of reaction products generated by the enzymatic activity of His-mATGL288-Str

As the first step, 2 mM of trioleoylglycerol as a substrate containing 225 μM PC:PI (3:1) (Sigma–Aldrich) or 0.6 mM dioleoylglycerol (mixture of 65% of 1,3-sn-DAG and 35% of 1,2-*rac*-DAG) containing 68 μM PC:PI (3:1) was dissolved in toluene and evaporated using a constant stream of gaseous nitrogen. The lipid micelles were prepared by sonication using the microtip MS 73 (output power 20% for 1 min with intervening breaks of similar length) in 0.1 M potassium phosphate buffer adjusted to pH 7.0. The substrate was adjusted to 5% (w/v) FA-free BSA. To determine the basal enzymatic hydrolytic activity of His-mATGL288-Str, 1 μg of purified protein in a total volume of 25 μl sucrose solution (250 mM sucrose, 1 mM EDTA, pH 7.0, and 1 mM DTT) was incubated for 60 min at 37 °C after adding the same volume of emulsified 2 mM trioleoylglycerol or DAG or MAG substrate. In case of the stimulated activity of His-mATGL288-Str, the purified protein was mixed in a 1:1 M ratio with SMT3-TEV-mCGI58-HIS. Basal and CGI-58–stimulated hydrolytic activities were inhibited by the addition of equimolar amounts of SMT3-TEV-hG0S2-HIS. As a blank measurement, 25 μl of sucrose solution were mixed with 25 μl of substrate and incubated under identical conditions. To analyze the reaction products, the reaction was stopped by transferring the samples into glass vials with 2.5 ml chloroform/methanol (2:1) containing 1% acetic acid. After the addition of 450 μl water, the samples were vortexed and centrifuged at 1000*g* for 10 min to induce phase separation. The upper phase was removed, and the lower phase was evaporated under a stream of nitrogen. Extracts were dissolved in chloroform/methanol (2:1), aliquots were applied onto TLC plates (silica gel 60 aluminum sheets; 20 × 20 cm; Merck) and separated with chloroform/acetone/acetic acid (90:8:1) until the solvent front reached 50% of the plate. The TLC plates were briefly dried and developed in toluene until the solvent front reached the top of the plate. The TLC plates were dried, immersed in a solution containing 10% H_3_PO_4_, 5% CuSO_4_, and 25% ethanol, and incubated at 120 °C for 20 min to visualize different lipid species.

To prepare 1-*rac*-monooleoyl glycerol containing 68 μM PC:PI (3:1), we followed the same procedure as described previously for trioleoylglycerol and dioleoylglycerol with some modifications. For lipid extraction, 1000 μl methyl *tert*-butyl ether (MTBE) extraction solution (MTBE:MeOH = 3:1 + 1% acetic acid) was used in the presence of 50 μl of water prior to phase separation by centrifugation. The upper phase was removed, and the lower organic phase was addressed to evaporation under the nitrogen. The dried-out extracts were resolubilized in 20 μl MTBE:MeOH = 3:1, and 10 μl of the extracts was spotted on TLC plate.

To study the effect of Atglistatin on ATGL, dioleoylglycerol was emulsified with 45 mM of PC:PI to a final concentration of 0.3 mM, and then added 5% (w/v) FA-free BSA. His-mATGL288-Str was preincubated with Atglistatin (kind gift from Rolf Breinbauer, Graz University of Technology) for 30 min. For the extraction, 1000 μl of Folch extraction solution (CHCl_3_:methanol:acetic acid, 66:33:1) were added, and phase separation was performed by centrifugation at 1000*g* for 10 min at 4 °C. An aliquot of 700 μl of the lower organic phase was evaporated under nitrogen gas, and the dried lipid extracts were solubilized in 40 μl CHCl_3_:methanol (2:1). Aliquots of 15 μl were spotted on TLC plate. Then the procedure was continued as described.

## Data availability

All data described are contained within this article and the supporting information.

## Supporting information

This article contains supporting information.

## Conflict of interest

The authors declare that they have no conflicts of interest with the contents of this article.
