# Supplementary material for: Optimized expression and purification of adipose triglyceride lipase improved hydrolytic and transacylation activities in vitro
Source: J Biol Chem. 2021 Sep 17;297(4):101206. doi: 10.1016/j.jbc.2021.101206 (PMC8506970; doi:10.1016/j.jbc.2021.101206)
Supplement: Supplemental Figs. S1–S3 and Tables S1 and S2 [file mmc1.docx]

**Supplementary Information of “Optimized expression and purification facilitate characterization of hydrolytic and transacylation activities of ATGL”**

**Table S1: List of mouse ATGL constructs designed for cloning and expression**

| **N/C term aa** | **Forward primer** | **Reverse primer** | **Design** | **Vector (pOPIN)** |
| --- | --- | --- | --- | --- |
| M1-Q257 | aagttctgtttcagggcccgTTCCCGAGGGAGACCAAGTGGAAC | ggtggctccagctagcTTGGTTCAGTAGGCCATTCCTCCTAAGG | His6-3C-POI-StrepII | pOPINFS |
| M1-E272 | aagttctgtttcagggcccgTTCCCGAGGGAGACCAAGTGGAAC | ggtggctccagctagcCTCTTCCTGGGGGACAACTGGGG | His6-3C-POI-StrepII | pOPINFS |
| M1-D288 | aagttctgtttcagggcccgTTCCCGAGGGAGACCAAGTGGAAC | ggtggctccagctagcATCCTCCTCTCCAGCCCTCTCCTCC | His6-3C-POI-StrepII | pOPINFS |
| M1-K295 | aagttctgtttcagggcccgTTCCCGAGGGAGACCAAGTGGAAC | ggtggctccagctagcTTTTCTATAAGGCTGCAATTGATCCTCCTCTC | His6-3C-POI-StrepII | pOPINFS |
| M1-D364 | aagttctgtttcagggcccgTTCCCGAGGGAGACCAAGTGGAAC | ggtggctccagctagcATCTTCAGGGACATCAGGCAGCCAC | His6-3C-POI-StrepII | pOPINFS |
| M1-H390 | aagttctgtttcagggcccgTTCCCGAGGGAGACCAAGTGGAAC | ggtggctccagctagcATGGTCACCCAATTTCCTCTTGGCC | His6-3C-POI-StrepII | pOPINFS |
| M1-C486 | aagttctgtttcagggcccgTTCCCGAGGGAGACCAAGTGGAAC | ggtggctccagctagcGCAAGGCGGGAGGCCAGGTG | His6-3C-POI-StrepII | pOPINFS |
| M1-Q257 | aagttctgtttcagggcccgTTCCCGAGGGAGACCAAGTGGAAC | atggtctagaaagctttaTTGGTTCAGTAGGCCATTCCTCCTAAGG | His6-GST-3C-POI | pOPINJ |
| M1-E272 | aagttctgtttcagggcccgTTCCCGAGGGAGACCAAGTGGAAC | atggtctagaaagctttaCTCTTCCTGGGGGACAACTGGG | His6-GST-3C-POI | pOPINJ |
| M1-D288 | aagttctgtttcagggcccgTTCCCGAGGGAGACCAAGTGGAAC | atggtctagaaagctttaATCCTCCTCTCCAGCCCTCTCCTCC | His6-GST-3C-POI | pOPINJ |
| M1-K295 | aagttctgtttcagggcccgTTCCCGAGGGAGACCAAGTGGAAC | atggtctagaaagctttaTTTTCTATAAGGCTGCAATTGATCCTCCTCTC | His6-GST-3C-POI | pOPINJ |
| M1-D364 | aagttctgtttcagggcccgTTCCCGAGGGAGACCAAGTGGAAC | atggtctagaaagctttaATCTTCAGGGACATCAGGCAGCCAC | His6-GST-3C-POI | pOPINJ |
| M1-H390 | aagttctgtttcagggcccgTTCCCGAGGGAGACCAAGTGGAAC | atggtctagaaagctttaATGGTCACCCAATTTCCTCTTGGCC | His6-GST-3C-POI | pOPINJ |
| M1-C486 | aagttctgtttcagggcccgTTCCCGAGGGAGACCAAGTGGAAC | atggtctagaaagctttaGCAAGGCGGGAGGCCAGGTG | His6-GST-3C-POI | pOPINJ |
| M1-Q257 | aagttctgtttcagggcccgTTCCCGAGGGAGACCAAGTGGAAC | atggtctagaaagctttaTTGGTTCAGTAGGCCATTCCTCCTAAGG | His6-MBP-3C-POI | pOPINM |
| M1-E272 | aagttctgtttcagggcccgTTCCCGAGGGAGACCAAGTGGAAC | atggtctagaaagctttaCTCTTCCTGGGGGACAACTGGGG | His6-MBP-3C-POI | pOPINM |
| M1-D288 | aagttctgtttcagggcccgTTCCCGAGGGAGACCAAGTGGAAC | atggtctagaaagctttaATCCTCCTCTCCAGCCCTCTCCTCC | His6-MBP-3C-POI | pOPINM |
| M1-K295 | aagttctgtttcagggcccgTTCCCGAGGGAGACCAAGTGGAAC | atggtctagaaagctttaTTTTCTATAAGGCTGCAATTGATCCTCCTCTC | His6-MBP-3C-POI | pOPINM |
| M1-D364 | aagttctgtttcagggcccgTTCCCGAGGGAGACCAAGTGGAAC | atggtctagaaagctttaATCTTCAGGGACATCAGGCAGCCAC | His6-MBP-3C-POI | pOPINM |
| M1-H390 | aagttctgtttcagggcccgTTCCCGAGGGAGACCAAGTGGAAC | atggtctagaaagctttaATGGTCACCCAATTTCCTCTTGGCC | His6-MBP-3C-POI | pOPINM |
| M1-C486 | aagttctgtttcagggcccgTTCCCGAGGGAGACCAAGTGGAAC | atggtctagaaagctttaGCAAGGCGGGAGGCCAGGTG | His6-MBP-3C-POI | pOPINM |
| M1-Q257 | aagttctgtttcagggcccgTTCCCGAGGGAGACCAAGTGGAAC | atggtctagaaagctttaTTGGTTCAGTAGGCCATTCCTCCTAAGG | His6-NUSA-3C-POI | pOPINNUSA |
| M1-E272 | aagttctgtttcagggcccgTTCCCGAGGGAGACCAAGTGGAAC | atggtctagaaagctttaCTCTTCCTGGGGGACAACTGGGG | His6-NUSA-3C-POI | pOPINNUSA |
| M1-D288 | aagttctgtttcagggcccgTTCCCGAGGGAGACCAAGTGGAAC | atggtctagaaagctttaATCCTCCTCTCCAGCCCTCTCCTCC | His6-NUSA-3C-POI | pOPINNUSA |
| M1-K295 | aagttctgtttcagggcccgTTCCCGAGGGAGACCAAGTGGAAC | atggtctagaaagctttaTTTTCTATAAGGCTGCAATTGATCCTCCTCTC | His6-NUSA-3C-POI | pOPINNUSA |
| M1-D364 | aagttctgtttcagggcccgTTCCCGAGGGAGACCAAGTGGAAC | atggtctagaaagctttaATCTTCAGGGACATCAGGCAGCCAC | His6-NUSA-3C-POI | pOPINNUSA |
| M1-H390 | aagttctgtttcagggcccgTTCCCGAGGGAGACCAAGTGGAAC | atggtctagaaagctttaATGGTCACCCAATTTCCTCTTGGCC | His6-NUSA-3C-POI | pOPINNUSA |
| M1-C486 | aagttctgtttcagggcccgTTCCCGAGGGAGACCAAGTGGAAC | atggtctagaaagctttaGCAAGGCGGGAGGCCAGGTG | His6-NUSA-3C-POI | pOPINNUSA |
| M1-Q257 | aagttctgtttcagggcccgTTCCCGAGGGAGACCAAGTGGAAC | atggtctagaaagctttaTTGGTTCAGTAGGCCATTCCTCCTAAGG | His6-SMT3-3C-POI | pOPINS3C |
| M1-E272 | aagttctgtttcagggcccgTTCCCGAGGGAGACCAAGTGGAAC | atggtctagaaagctttaCTCTTCCTGGGGGACAACTGGGG | His6-SMT3-3C-POI | pOPINS3C |
| M1-D288 | aagttctgtttcagggcccgTTCCCGAGGGAGACCAAGTGGAAC | atggtctagaaagctttaATCCTCCTCTCCAGCCCTCTCCTCC | His6-SMT3-3C-POI | pOPINS3C |
| M1-K295 | aagttctgtttcagggcccgTTCCCGAGGGAGACCAAGTGGAAC | atggtctagaaagctttaTTTTCTATAAGGCTGCAATTGATCCTCCTCTC | His6-SMT3-3C-POI | pOPINS3C |
| M1-D364 | aagttctgtttcagggcccgTTCCCGAGGGAGACCAAGTGGAAC | atggtctagaaagctttaATCTTCAGGGACATCAGGCAGCCAC | His6-SMT3-3C-POI | pOPINS3C |
| M1-H390 | aagttctgtttcagggcccgTTCCCGAGGGAGACCAAGTGGAAC | atggtctagaaagctttaATGGTCACCCAATTTCCTCTTGGCC | His6-SMT3-3C-POI | pOPINS3C |
| M1-C486 | aagttctgtttcagggcccgTTCCCGAGGGAGACCAAGTGGAAC | atggtctagaaagctttaGCAAGGCGGGAGGCCAGGTG | His6-SMT3-3C-POI | pOPINS3C |
| M1-Q257 | aagttctgtttcagggcccgTTCCCGAGGGAGACCAAGTGGAAC | atggtctagaaagctttaTTGGTTCAGTAGGCCATTCCTCCTAAGG | His6-TRX-3C-POI | pOPINTRX |
| M1-E272 | aagttctgtttcagggcccgTTCCCGAGGGAGACCAAGTGGAAC | atggtctagaaagctttaCTCTTCCTGGGGGACAACTGGGG | His6-TRX-3C-POI | pOPINTRX |
| M1-D288 | aagttctgtttcagggcccgTTCCCGAGGGAGACCAAGTGGAAC | atggtctagaaagctttaATCCTCCTCTCCAGCCCTCTCCTCC | His6-TRX-3C-POI | pOPINTRX |
| M1-K295 | aagttctgtttcagggcccgTTCCCGAGGGAGACCAAGTGGAAC | atggtctagaaagctttaTTTTCTATAAGGCTGCAATTGATCCTCCTCTC | His6-TRX-3C-POI | pOPINTRX |
| M1-D364 | aagttctgtttcagggcccgTTCCCGAGGGAGACCAAGTGGAAC | atggtctagaaagctttaATCTTCAGGGACATCAGGCAGCCAC | His6-TRX-3C-POI | pOPINTRX |
| M1-H390 | aagttctgtttcagggcccgTTCCCGAGGGAGACCAAGTGGAAC | atggtctagaaagctttaATGGTCACCCAATTTCCTCTTGGCC | His6-TRX-3C-POI | pOPINTRX |
| M1-C486 | aagttctgtttcagggcccgTTCCCGAGGGAGACCAAGTGGAAC | atggtctagaaagctttaGCAAGGCGGGAGGCCAGGTG | His6-TRX-3C-POI | pOPINTRX |

**Table S2: Primers required for the cloning of pST50-Trc4-SMT3-TEV-GGG-mATGL-Str**

| **Variant** | **Forward primer** | **Reverse primer** | **vector** |
| --- | --- | --- | --- |
| pST50_Trc4_ATGL | aaggagatatacatatgggatccATGTCGGACTCAGAAGTCAATC | ccgccaccACCAATCTGTTCTCTGTGAGC | pST50 |
| pST50_Trc4_SMT3 | cagattggtggtggcggagaaaacctgtattttcagggcATGTTTCCACGC GAAACCAAATGGAAC | tggctccaagcgctgccggcGCACGGAGGCAGGCCTGG | pST50 |

**
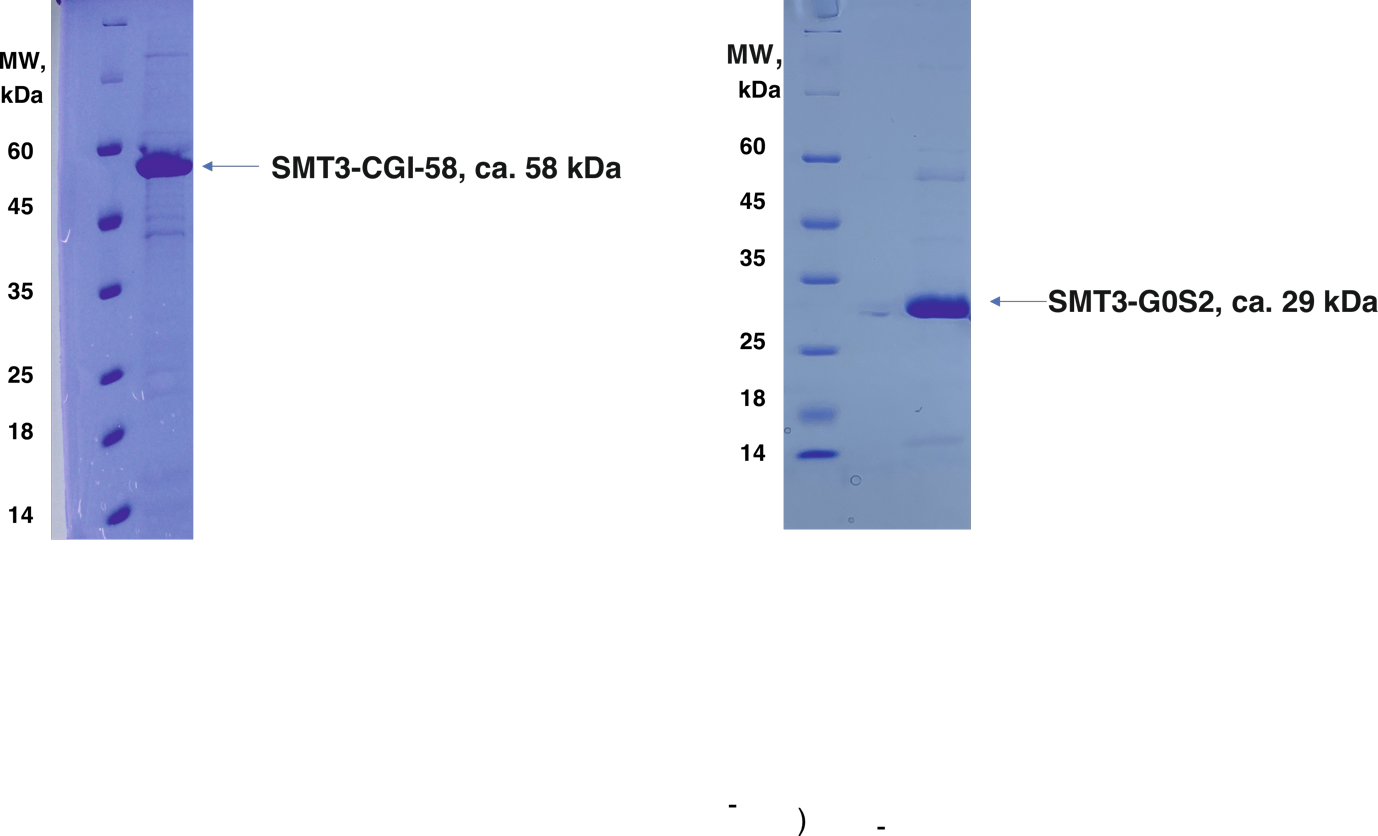
**

**Figure S1.** SDS-PAGE of purified proteins used for co-activation/inhibition of ATGL288: SMT3-CGI-58 (left), SMT3-G0S2 (right).


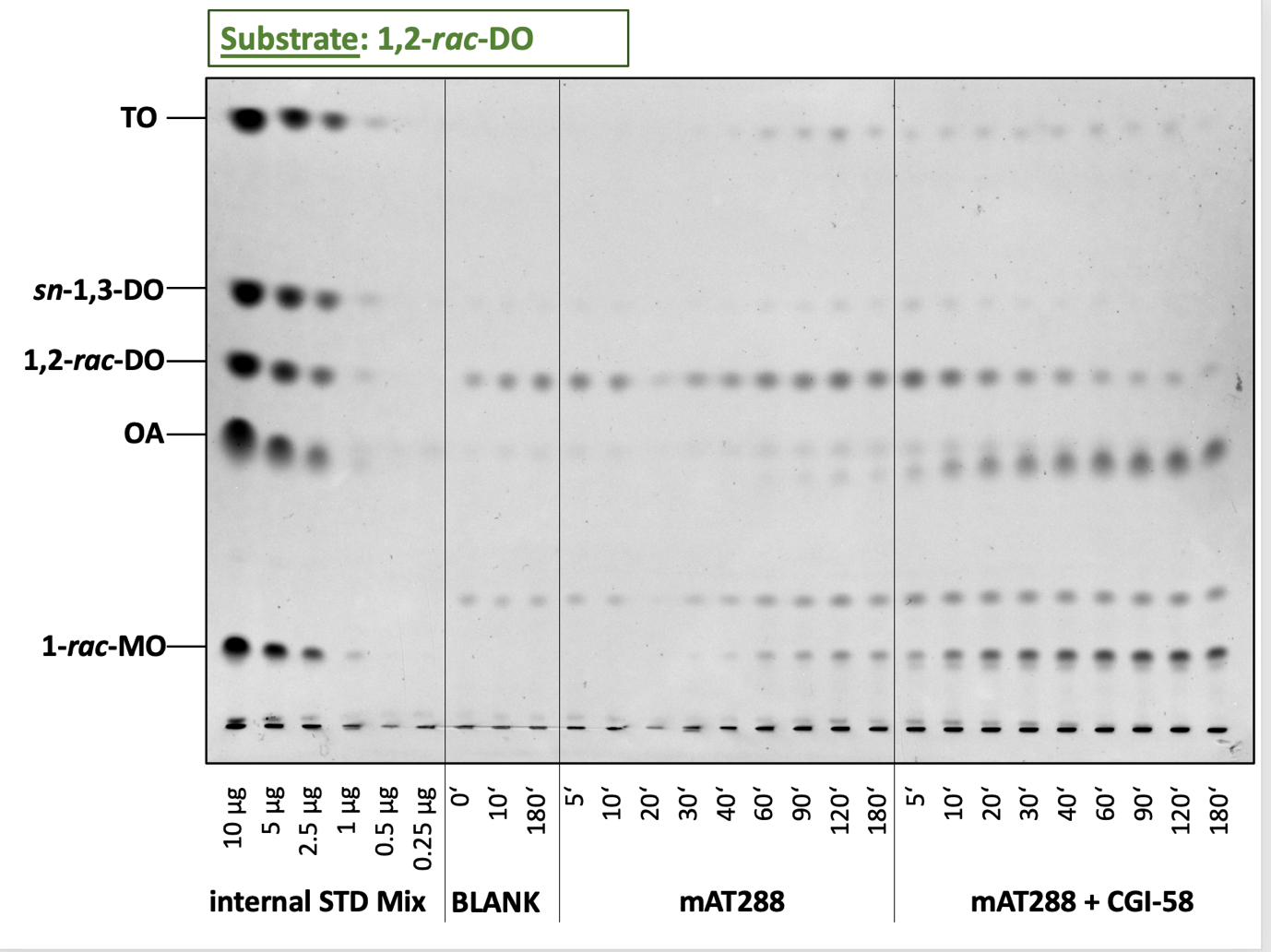


**Figure S2.** Thin-layer chromatography showing triacylglycerol build-up with 1,2-rac diacylglycerol (DAG) as substrate indicates DAG hydrolysis and acyl-CoA independent transacylation. Basal and CGI-58 stimulated enzymatic activity of purified His-mATGL288-Str presented at different time points.


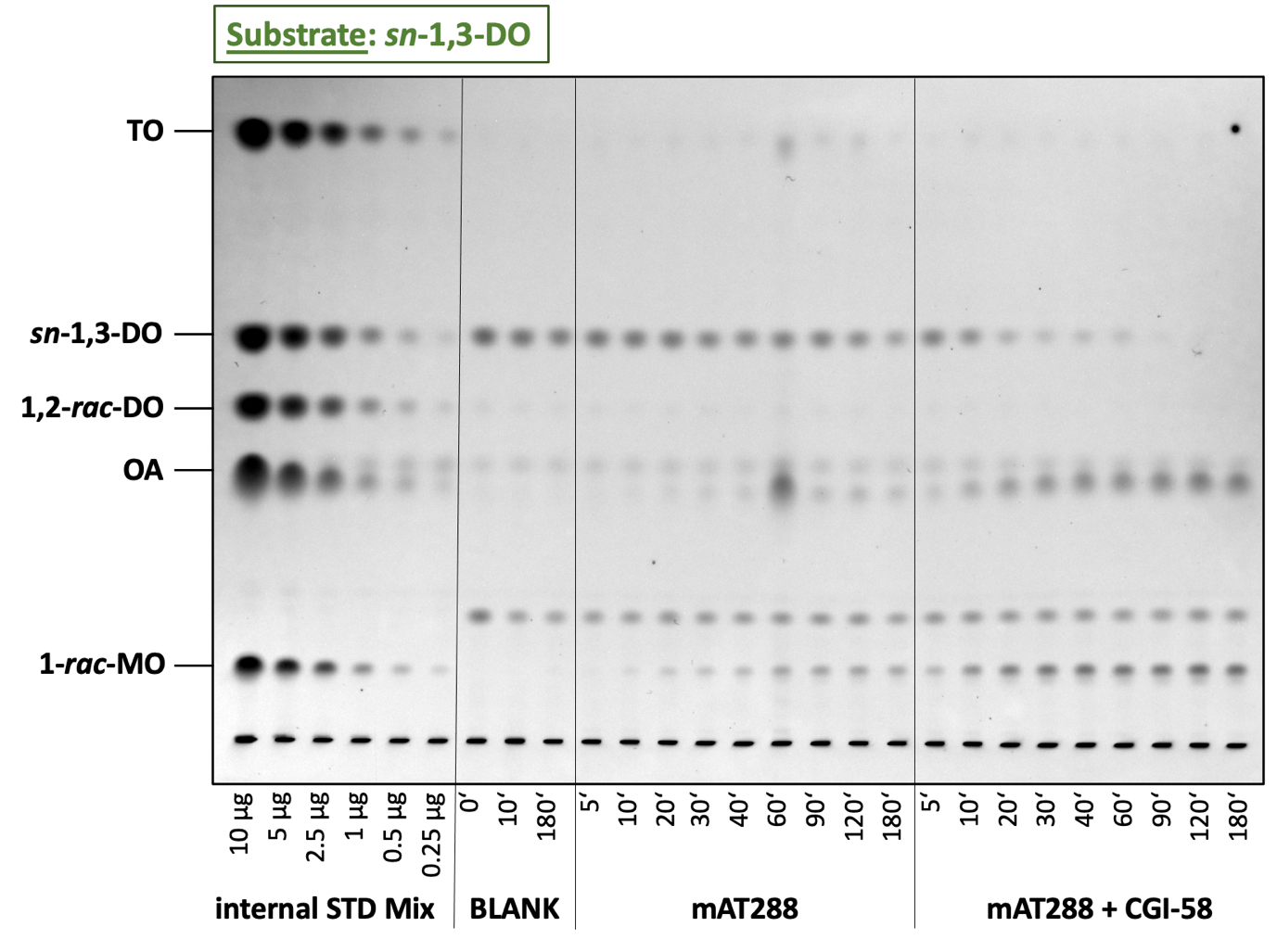


**Figure S3.** Thin-layer chromatography showing triacylglycerol build-up with 1,3-sn diacylglycerol (DAG) as substrate indicates DAG hydrolysis and acyl-CoA independent transacylation. Basal and CGI-58 stimulated enzymatic activity of purified His-mATGL288-Str presented at different time points.
